# Hypoxic extracellular vesicles from hiPSCs protect cardiomyocytes from oxidative damage by transferring antioxidant proteins and enhancing Akt/Erk/NRF2 signaling

**DOI:** 10.1186/s12964-024-01722-7

**Published:** 2024-07-09

**Authors:** Sylwia Bobis-Wozowicz, Milena Paw, Michał Sarna, Sylwia Kędracka-Krok, Kinga Nit, Natalia Błażowska, Anna Dobosz, Ruba Hammad, Toni Cathomen, Ewa Zuba-Surma, Małgorzata Tyszka-Czochara, Zbigniew Madeja

**Affiliations:** 1 Faculty of Biochemistry, Biophysics and Biotechnology, Department of Cell Biology, Jagiellonian University, Kraków, Poland; 2 Faculty of Biochemistry, Biophysics and Biotechnology, Department of Biophysics, Jagiellonian University, Krakow, Poland; 3 Faculty of Biochemistry, Biophysics and Biotechnology, Department of Physical Biochemistry, Jagiellonian University, Krakow, Poland; 4 Freiburg iPS Core Facility, Institute for Transfusion Medicine and Gene Therapy, Medical Center- University of Freiburg, Freiburg, Germany; 5 Center for Chronic Immunodeficiency (CCI), University of Freiburg, Freiburg, Germany; 6 Faculty of Pharmacy, Department of Food Chemistry and Nutrition, Jagiellonian University Medical College, Kraków, Poland

**Keywords:** Cardiovascular disease, Heart regeneration, Extracellular vesicles, Induced pluripotent stem cells, Hypoxia, Cardiomyocyte, NRF2, Cell survival, Antioxidant response

## Abstract

**Background:**

Stem cell-derived extracellular vesicles (EVs) are an emerging class of therapeutics with excellent biocompatibility, bioactivity and pro-regenerative capacity. One of the potential targets for EV-based medicines are cardiovascular diseases (CVD). In this work we used EVs derived from human induced pluripotent stem cells (hiPSCs; hiPS-EVs) cultured under different oxygen concentrations (21, 5 and 3% O_2_) to dissect the molecular mechanisms responsible for cardioprotection.

**Methods:**

EVs were isolated by ultrafiltration combined with size exclusion chromatography (UF + SEC), followed by characterization by nanoparticle tracking analysis, atomic force microscopy (AFM) and Western blot methods. Liquid chromatography and tandem mass spectrometry coupled with bioinformatic analyses were used to identify differentially enriched proteins in various oxygen conditions. We directly compared the cardioprotective effects of these EVs in an oxygen-glucose deprivation/reoxygenation (OGD/R) model of cardiomyocyte (CM) injury. Using advanced molecular biology, fluorescence microscopy, atomic force spectroscopy and bioinformatics techniques, we investigated intracellular signaling pathways involved in the regulation of cell survival, apoptosis and antioxidant response. The direct effect of EVs on NRF2-regulated signaling was evaluated in CMs following NRF2 inhibition with ML385.

**Results:**

We demonstrate that hiPS-EVs derived from physiological hypoxia at 5% O_2_ (EV-H5) exert enhanced cytoprotective function towards damaged CMs compared to EVs derived from other tested oxygen conditions (normoxia; EV-N and hypoxia 3% O_2_; EV-H3). This resulted from higher phosphorylation rates of Akt kinase in the recipient cells after transfer, modulation of AMPK activity and reduced apoptosis. Furthermore, we provide direct evidence for improved calcium signaling and sustained contractility in CMs treated with EV-H5 using AFM measurements. Mechanistically, our mass spectrometry and bioinformatics analyses revealed differentially enriched proteins in EV-H5 associated with the antioxidant pathway regulated by NRF2. In this regard, EV-H5 increased the nuclear translocation of NRF2 protein and enhanced its transcription in CMs upon OGD/R. In contrast, inhibition of NRF2 with ML385 abolished the protective effect of EVs on CMs.

**Conclusions:**

In this work, we demonstrate a superior cardioprotective function of EV-H5 compared to EV-N and EV-H3. Such EVs were most effective in restoring redox balance in stressed CMs, preserving their contractile function and preventing cell death. Our data support the potential use of hiPS-EVs derived from physiological hypoxia, as cell-free therapeutics with regenerative properties for the treatment of cardiac diseases.

**Supplementary Information:**

The online version contains supplementary material available at 10.1186/s12964-024-01722-7.

## Background

Cardiovascular disease (CVD) remains a prominent health issue worldwide, wherein ischemic heart disease is responsible for the highest mortality rates in developed countries [1]. The unsatisfactory clinical improvement of patients results in a serious socio-economic burden. The present medical treatments mainly comprise cardiac surgery and pharmacological drugs that restrict reperfusion injury. However, neither is sufficient to restore the function of the contractile cells of the heart - the cardiomyocytes (CMs). Given the complex architecture of the human heart, which is composed of different cell types with diverse functions, such as endothelial cells, immune cells and fibroblasts, their perfectly orchestrated communication with CMs determines the proper functioning of the entire organ. [2]. However, the delicate balance of various stimuli within the heart tissue can be disrupted in cases of acute or chronic CVD. CMs are vulnerable to damage from hypoxia or oxidative injury, as in the case of postoperative reoxygenation [3]. Damaged CMs are then removed and replaced by fibroblasts during the natural healing process of the heart. Unfortunately, fibroblasts eventually form a non-functional fibrotic scar that can ultimately lead to heart failure [4]. Thus, there is an urgent need to develop effective therapies that safeguard the heart from CM loss.

One of emerging classes of biologically active therapeutics are extracellular vesicles (EVs). These small, membranous vesicles are released by various cell types, including stem cells, under steady-state and stressful conditions [5]. The EV family encompasses particles of varying sizes (30–1000 nm), origin, and molecular composition that are determined by the type and status of their parental cell. EVs can be classified into exosomes, which originate from the endosomal compartment, microvesicles released from the plasma membrane, and apoptotic bodies formed during programmed cell death (apoptosis) [6]. They can transfer bioactive molecules, such as proteins, nucleic acids, and lipids to recipient cells, therefore, they are crucial regulators of intercellular communication [7]. In recent years, EVs have attracted considerable attention in the field of regenerative medicine, demonstrating efficacy in cardioprotection and heart regeneration. In this context, EVs have shown potential to mitigate damage caused by CVD and to protect the heart from ischemic injury [8, 9]. Reparative abilities have been demonstrated for EVs obtained from various cell sources, such as mesenchymal stromal cells (MSCs), pluripotent stem cells (PSCs), endothelial cells, cardiac progenitor cells (CPCs) or cardiospheres, making them appealing for clinical use. EV-based therapeutics have proven the ability to enhance CMs activity, reduce inflammation, promote angiogenesis and reduce cell death in both cellular and animal models of heart disease [9].

Studies conducted by our group and others have demonstrated the enhanced pro-angiogenic and cardio-protective effects of EVs derived from PSCs, including embryonic stem cells (ESCs) [10] and induced pluripotent stem cells (iPSCs; iPS-EVs) [11–14]. In this work, we sought to identify optimal conditions for the culture of human iPSCs, in order to enhance the therapeutic potential of hiPS-EVs. As PSCs naturally reside in hypoxic niches, a condition that supports the expression of pluripotency markers and affects cellular metabolism [15], we verified the influence of oxygen concentration on the cytoprotective function of hiPS-EVs. Of significance, an alteration in oxygen level within the cell microenvironment was shown to affect not only EV biogenesis but also their molecular composition and, subsequently, their functional attributes [16, 17]. Studies have shown that low-oxygen environments with 1–5% availability increase the pro-angiogenic and pro-proliferative properties of EVs derived from different cell types [18–21]. In our recent study, we found that exposure of hiPSCs to 5% hypoxia enhances antifibrotic activity of hiPS-EVs in vitro and in vivo and resulted in amelioration of cardiac fibrosis [22]. However, direct evidence of cardio-protective effects of hiPS-EVs from different oxygen concentrations on CMs and underlying molecular mechanisms is still lacking.

To address this research gap, we conducted a thorough analysis evaluating the effects of hiPS-EVs obtained from varying oxygen concentrations, on CMs subjected to oxygen-glucose deprivation, followed by reoxygenation (OGD/R). We tested EVs obtained from normoxic (atmospheric; EV-N) and from hypoxic conditions at 5% and 3% O_2_ (designated as EV-H5 and EV-H3 respectively). In vitro models of CMs injury are often used as a surrogate for cardiac injury caused by ischemia and reperfusion, which helps to define new targets for the treatment of CVDs [23]. Human CMs provide an advantage over animal models, as they possess unique biochemical and physiological features that differ from animal cells [24]. At the cellular level, cardiomyocytes in the OGD/R model are progressively damaged by extensively produced reactive oxygen species (ROS). An elevated level of ROS results in the oxidation of proteins, lipids, and DNA, exceeding a cell’s defense capabilities and leading to cell death. A critical role in anti-oxidant defense mechanisms play the nuclear factor (erythroid-derived 2)-like 2 (NFE2L2) also known as NRF2. This transcription factor (TF) regulates the expression of various enzymes and molecules that possess anti-oxidative and detoxifying properties. These include heme oxygenase 1 (HO-1, encoded by *HMOX-1* gene), catalase (CAT), superoxide dismutase 2 (SOD2), peroxiredoxins (PRDXs), and glutathione S-transferases (GSTs) [25]. NRF2-mediated signaling cooperates with anti-apoptotic and pro-survival pathways regulated by intracellular kinases such as protein kinase B (PKB, also known as Akt) and mitogen-activated protein kinases (MAPK), including extracellular signal-regulated kinase 1/2 (Erk1/2) [26]. Activation of anti-oxidant and pro-survival pathways in CMs was shown to be responsible for the cardioprotective function of EVs derived from native or engineered MSCs overexpressing NRF2 [27–30]. However, the direct effect of EVs on the mechanical properties of human CMs, including their contractile capacity, has not yet been demonstrated. In this study, we not only investigated the molecular signature and basic functions of CMs subjected to hiPS-EV treatment after OGD/R, but we also examined the mechanical properties of these cells using a cutting-edge technology based on atomic force microscopy (AFM).

## Methods

### Cell culture

Three iPSC cell lines were used in this study, as described before [22]. The cell lines were generated with integration-free reprograming systems and were designated as: L1 - generated in our laboratory from umbilical cord-derived MSCs by transduction with Sendai viral vectors (OSKM) [11]; L2 - episomal hiPSC line purchased from Gibco (A18945) and generated from CD34-positive blood cells (OSKM, NANOG, Lin28, SV40); L3 - generated in the laboratory of Prof. Toni Cathomen - University of Freiburg - Medical Center, Frieburg, Germany, from foreskin fibroblasts by transduction with Sendai viral vectors (OSKM). IPSCs were cultured in serum-free, feeder-free and xeno-free conditions, in Essential8 medium (Gibco/Thermo Fisher Scientific), in the presence of antibiotics (penicillin/streptomycin; P/S; Gibco) in cell culture dishes covered with human recombinant vitronectin (Gibco), in a standard cell incubator (21% O_2_) or under hypoxic conditions: 5% O_2_ (H5) or 3% O_2_ (H3), in an InvivO2 chamber (Ruskinn). Cells were gradually adapted to hypoxia by sequentially decreasing the oxygen concentration by 3% per day. Cells were passaged every 4 days by detachment from cell culture vessels with 50 µM EDTA solution (Gibco). ROCK kinase inhibitor (Y-27,632; Millipore) was added to the cells at a concentration of 10 µM for the first 24 h after passage. The cell medium was changed daily.

Human dermal fibroblasts (hDF; ATCC) were cultured in Dulbecco’s modified Eagle’s medium/high glucose (DMEM/HG; Sigma-Aldrich/Merck) supplemented with 10% fetal bovine serum (FBS; Gibco) and antibiotics (P/S). Cell cultures were carried out in the presence of 5% CO_2_ and 80–90% humidity.

### Differentiation of hiPSCs into cardiomyocytes

hiPSC-L3 was differentiated into cardiomyocytes (CMs) by modulation of Wnt-β-catenin pathway, based on a previously published protocol [31]. Briefly, 8 × 10e4 cells/well were seeded on a 12-well plate coated with Geltrex, LDEV-free, reduced growth factor basement membrane matrix (Gibco) in Essential8 medium. Three days after plating, at day 0 of differentiation, the cells were treated with 12µM GSK3β inhibitor (Merck) in RPMI-1640 medium (Sigma) containing 2% B27 supplement without insulin (Gibco) (RPMI/B27-ins) for 24 h. The medium was then removed and replaced with fresh medium for the next 48 h. On day 3 of differentiation, the cells were stimulated with 5 µM IWR-1 (Merck) in RPMI/B27-ins for 48 h. From day 7 of the process, the medium was changed to RPMI-1640 medium with 2% B27 supplement with insulin (Gibco) (RPMI/B27 + ins). After 15 days of differentiation, the cells were split in a 1:4 ratio and replated into new Geltrex-coated plates in RPMI/B27 + ins supplemented with 20% FBS. FBS was gradually removed by changing the medium by half daily for 3 days. Next, cells were subjected to metabolic selection with sodium lactate (4 mM; Sigma) [32] in DMEM without glucose (Gibco) for 6 days to obtain pure cultures of CMs. Such cells were used in the experiments.

### Isolation of EVs

To isolate EVs, hiPSCs were first adapted to each experimental oxygen condition for at least 4 passages. Each batch of EVs was isolated from approximately 100 ml of conditioned media harvested from iPSCs cultured at 70–90% cell density using ultrafiltration (UF) combined with size exclusion chromatography (SEC) [22]. The media were first centrifuged at 500 × g, 8 min, then at 2,000 × g, 15 min. They were then concentrated in 15 ml Amicon (Merck) tubes with a protein cut-off of 10 kDa and applied to the 70qEV (Izon) columns. The EV-containing fractions (volume 1.5 ml after the void volume of the first 3 ml) were collected in 1.5 ml low-adhesion tubes. Next, they were concentrated in 4 ml Amicon tubes with a protein cut-off of 10 kDa. All isolation steps were performed at 4^o^C. To isolate EVs from hDF, cells were grown at passage 4–6 to 70–90% confluence, then washed twice with PBS before adding collection medium consisting of DMEM/HG containing 0.1% bovine serum albumin (BSA) and P/S. The conditioned medium was collected 48 h later and processed with the UF-10 kDa + SEC method as described above. EV preparations were stored in aliquots at -80^o^C until use.

### Nanoparticle tracking analysis

EV size and concentration were analyzed using the NanoSight instrument (Malvern). EV samples were diluted 1:1000 in PBS. Measurements were taken using the following parameters: camera level: 11, detection threshold: 5. Each sample was recorded for 60 s. Three preparations were analyzed for each EV type.

### EV visualization

EVs were visualized using atomic force microscopy [33]. Prior to analysis, a small droplet of EVs suspended in PBS was placed on a freshly cleaved mica surface and allowed to adhere for 30 min at room temperature (RT). The EVs were imaged in Tapping AC mode at RT using a Bruker Bioscope Catalyst AFM.

### Liquid chromatography and mass spectrometry (LC-MS/MS)

#### Sample preparation for LC-MS/MS analysis

After EV collection in lysis buffer (2% SDS 0.1MTris pH = 7.5), samples were sonicated for 15 min at 320 W (intensity set to high) with a 30 s/30 s ON/OFF time interval using a Bioruptor UCD-200 sonicator (Diagenode, Liège, Belgium). Samples were then incubated at 95^o^C for 5 min and centrifuged (20,000 × g, 10 min, RT). The supernatant was stored for LC-MS/MS analysis and was prepared using the Filter Aided Sample Preparation (FASP) method [34]. Briefly, the supernatant was diluted with 8 M urea in 50 ammonium bicarbonate (300 µl), reduced with DTT (final concentration was 50 mM, 15 min) and applied to the 30 kDa cut-off filter (Vivacon 500, Sartorius Stedim, Germany). After centrifugation (14,000 × g, twice 15 min, RT), the proteins were washed with 200 µl of 8 M urea and centrifuged (14,000 × g, twice 45 min, RT). The proteins were alkylated with iodoacetamide (final concentration 0.1 mg/ml in 8 M urea, 20 min, in the dark). The samples were centrifuged and then washed three times with 8 M urea (14,000 × g, two times 25 min, RT) and four times with 50 mM ammonium bicarbonate (14,000 × g, two times 20 min, RT). After the last centrifugation step, trypsin in ammonium bicarbonate was added (protein to trypsin ratio was 100:1 w/w). After protein digestion on the filter (overnight, 37°C), the resulting peptides were spun down (14,000 × g, 30 min, 25°C) and the filter unit was washed twice with 40 µl ammonium bicarbonate and additionally with 50 µl 0.5 M NaCl (14,000 × g, 30 min, 25°C). After the addition of 2 µl 100% TFA, the peptide samples were centrifuged (35,000 × g, 20 min, 4°C) and transferred to a vial insert prior to LC-MS/MS analysis.

#### Liquid chromatography and tandem mass spectrometry (LC-MS/MS) measurement

Peptides were analyzed using a Q Exactive high resolution mass spectrometer (Thermo Fisher Scientific) with DPV-550 Digital PicoView nanospray source coupled to nanoHPLC and an UltiMate 3000RS LC nanoSystem (Dionex).Peptides were applied to a C18 precolumn (Acclaim PepMap Nano trap Column) using 2% acetonitrile with 0. 05% TFA as mobile phase and further separated on a 50 cm × 75 μm RP column (Acclaim PepMap 75 μm 100 Å Nano Series TM Column) with a gradient of 2–40% ACN in 0.05% FA for 240 min. Full MS scans were acquired in the Orbitrap mass analyzer over the m/z 300–2000 range with a resolution of 70,000 (at m/z 200). The twelve most intense peaks with charge state ≥ 2 were fragmented in the HCD collision cell at a normalized collision energy of 27% (the isolation window was 1.2 m/z). The tandem mass spectrum was acquired in the Orbitrap mass analyzer with a resolution of 17,500 at m/z 200.

#### LC-MS/MS data analysis

Raw LC-MS/MS files were analyzed using MaxQuant 2.1.4.0 and an Andromeda server against the SwissProt database with Homo sapiens taxonomy restriction (20,404 sequences), supplemented by the Common Protein Contamination database. LFQ intensity and iBAQ quantification (Σ intensity/# theoretical peptides) were enabled and standard software settings were used, including a false discovery rate (FDR) of less than 1% for peptide and protein identification. Search parameters were as follows: enzyme - trypsin; number of missed cleavages − 2; static modification - carbamidomethylation (C); dynamic modifications - oxidation (M) and acetyl (protein N-terminal). Statistical analysis was performed using Perseus software 1.6.7.0. Protein groups from the reverse database, common protein contaminants and proteins identified by site only were filtered out. Proteins detected based on at least two peptides were included in the analysis. Differential protein expression analysis in EVs derived from different oxygen conditions included proteins present in at least 6 out of 9 samples. Differentially expressed proteins based on t-test (*p* < 0.05) were selected for pathway enrichment analysis. Volcano plots were generated using the VolcaNoseR web application with *p* < 0.05 and FC > 1.3 [35]. Enriched pathways were identified using ReactomePA package in R [36] separately for up- and down-regulated proteins in each contrast (H3_N; H3_H5; H5_N), with *p* value < 0.1, q value < 0.05. Representative enriched pathways were selected and visualized in chord plots using the GOplot package in R [37]. Further analysis was performed using the Ingenuity Pathway Analysis (IPA, QIAGEN) software, which included differential proteins with *p* < 0.05 and exhibiting at least a 1.3-fold change. Only canonical pathways that met the Benjamini-Hochberg correction with a significance level of 0.05 are presented.

For iBAQ analysis of 5% most abundant proteins in each EV type, only proteins present in all replicates and identified based on at least 2 peptides in each sample and having iBAQ value in at least 2 samples in a group were included in the analysis. Protein-protein interaction (PPI) networks were then generated using STRING software [38], and pathways were enriched in STRING based on WIKIPathways [39], with FDR threshold set at 0.05.

### EV uptake by CMs

EV uptake by hiPS-CMs was analyzed by scanning laser confocal microscopy (Zeiss LSM 900 with Airyscan 2). First, cells were stained with DiO lipid probe (3,3́ -dioctadecyloxacarbocyanine, perchlorate; Invitrogen) according to the manufacturer’s protocol. In turn, EV-H5 were stained using 5 µM Vybrant DiD cell labeling solution (1,1’-dioctadecyl-3,3,3’,3’-tetramethylindodicarbocyanine, 4-chlorobenzenesulfonate salt; Invitrogen). Staining was performed at 37^o^C for 30 min, after which EVs were purified by SEC using qEV-70 columns (Izon). The purified EVs were then concentrated in 2 ml filter tubes with a 10 kDa protein cut-off (Amicon) and co-incubated with CMs for 30 min before imaging. High-resolution images were taken using scanning laser confocal microscopy (Zeiss LSM 900 with Airyscan 2), as descried before [22].

### Oxygen-glucose deprivation/reoxygenation (OGD/R) model of CM oxidative damage

CMs seeded on Geltrex-coated plates in RPMI/B27 + ins were subjected to hypoxia and reoxygenation in vitro. For this purpose, the cells were incubated in an ischemic buffer (137 mM NaCl, 15.8 mM KCl, 0.49 mM MgCl_2_, 0.9 mM CaCl_2_, 4 mM HEPES, 10 mM 2-deoxyglucose, 20 mM sodium lactate, 1 mM sodium dithionite, pH 6.3) [40], in 1% O_2_ obtained in a hypoxic chamber (InVivo2; Ruskinn). After 2 h of hypoxia, the cells were rapidly oxygenated outside the hypoxic chamber and the ischemic buffer was changed to a fresh medium RPMI/B27 + ins, supplemented or not with hiPS-EVs derived from different oxygen conditions (EV-N; EV-H5, EV-H3) at a concentration of 2.5 × 10e4 EV particles/cell, as previously established for cardiac fibroblasts [22]. EVs from three hiPSC lines were used together with EVs from DF in each experiment. Cells not exposed to OGD/R and not treated with EVs served as control. Cell assays were performed at 4 or 24 h after reoxygenation, as indicated.

### Measurement of ROS levels

ROS production was measured in CMs subjected to OGD/R with or without treatment with EVs using the CellROX Deep Red Reagent for oxidative stress detection (Invitrogen) according to the manufacturer’s recommendations. Briefly, 5 × 10e3 cells were plated into Geltrex-coated 96-well plates in RPMI/B27 + ins medium. The next day, cells were treated with OGD/R and 4 h after reoxygenation they were stained with CellROX solution (5 µM) for 30 min. After two washes with PBS, nuclei were stained with Hoechst 33258 solution (1 µg/ml; Sigma-Aldrich). Four images were taken for each experimental condition using the tile-scan method and a Leica DMI6000B fluorescence microscope (Leica). Fluorometric analysis was performed using ImageJ software (NIH, Bethesda, MD, USA). Data were calculated as the ratio of the mean fluorescence intensity detected in the red channel to the fluorescence intensity of DNA [41]. Results are presented as relative fluorescence intensity in experimental samples calibrated to control cells.

### Analysis of cell metabolism

The metabolic activity of CMs was assessed using the Cell Counting Kit-8 (Sigma-Aldrich). Briefly, cells were seeded on Geltrex-coated 96-well plates (5 × 10e3 cells per well) 24 h before the experiment. The cells were then subjected to OGD/R treatment and stimulation with EVs (2.5 × 10e4 EV particles/cell) for 24 h. After this time, the medium was changed into a fresh medium and the CCK-8 reagent was added for 2 h. Cellular metabolic activity was measured in an Infinite M200 Microplate Reader (Tecan), relative to control.

### Analysis of apoptosis

The percentage of live/dead/apoptotic cells was measured using the Apoptosis/Necrosis Detection Kit (Abcam), where live cells are stained blue, apoptotic cells are stained green and necrotic cells are stained red. The procedure was performed according to the protocol provided in the kit. Briefly, 4 × 10e4 CMs were seeded into Geltrex-coated glass-bottom 96 black-well plates (Eppendorf) one day prior to experiment. After 2 h of hypoxia 1% O_2_, the cells were treated with hiPS-EVs (2.5 × 10e4 EV particles/cell) for 24 h. Then the medium was removed, the cells were washed with PBS and incubated with Apopxin Green Solution, 7-AAD and CytoCalcein Violet 450 for 30 min. After washing with PBS (2x), cells were visualized in three fluorescence channels using the JuLi Stage Cell Imaging System (NanoEntek): GFP, RFP and DAPI, using 4 × 4 binning mode and autofocus. Cells present in each channel were counted by a blinded investigator.

### Quantitative real-time PCR (qPCR)

Total RNA was isolated from cells using the GeneMATRIX Universal RNA/miRNA Purification Kit (EURx). RNA concentration and purity were assessed using a spectrophotometer (Implen). Subsequently, 1–2 µg of RNA was used for cDNA synthesis using the NG dART RT-PCR Kit (EURx) according to the manufacturer’s instructions. Gene expression levels were analyzed by real-time quantitative PCR (qPCR) using the SybrGreen dye (Applied Biosystems/Thermo Fisher Scientific) and specific primer pairs (listed in Table 1). The reaction was performed on the 7500 Fast Real-Time PCR System (Applied Biosystems) under the following conditions: 50°C–2 min; 95°C–10 min; and 40 cycles: 95°C–15 s; 60°C–1 min. Data were calculated using the ∆∆Ct method and β-2-microglobulin as an endogenous control.

**Table 1:** Primer sequences used for qPCR

Gene name	Primer forward (5’ – 3’)	Primer reverse (5’ – 3’)
*β-2-microglobulin*	AATGCGGCATCTTCAAACCT	TGACTTTGTCACAGCCCAAGATA
*GATA4*	AACGACGGCAACAACGATAAT	GTTTTTTCCCCTTTGATTTTTGATC
*TNNT2*	AGACAGAGCGGAAAAGTGGG	CTCCTTGGCCTTCTCCCTCA
*OCT4*	CCTTCGCAAGCCCTCATTTCA	CCCACAGAACTCATACGGCG
*NANOG*	ACCTCAGCTACAAACAGGTGAAG	TTCTGCGTCACACCATTGCT
*HMOX1*	TCTTGGCTGGCTTCCTTACC	GGATGTGCTTTTCGTTGGGG
*SOD2*	GTTGGTGTCCAAGGCTCAGGTT	GTAAGCGTGCTCCCACACATCA
*CAT*	AGGGGCCTTTGGCTACTTTGAG	GAACCCGATTCTCCAGCAACAG
*GSTP1*	GGACCTCCGCTGCAAATACA	ACAGCAGGGTCTCAAAAGGC
*TNFα*	CCTCTGATGGCACCACCAG	TCTTCTCGAACCCCGAGTGA
*IL1β*	AGACATCACCAAGCTTTTTTGCT	GCACGATGCACCTGTACGAT
*IL6*	TTCGGCAAATGTAGCATG	AATAGTGTCCTAACGCTCATAC
*TLR4*	TTTCACCTGATGCTTCTTGCT	TCCTTACCCAGTCCTCATCCT
*BAX*	TTTTGCTTCAGGGTTTCATCCAG	CGGAAAAAGACCTCTCGGGG
*BCL-2*	AGATTGATGGGATCGTTGCCT	AGTCTACTTCCTCTGTGATGTTGT
*NRF2*	CACATCCAGTCAGAAACCAGTGG	GGAATGTCTGCGCCAAAAGCTG

### NRF2 inhibition

CMs subjected to OGD/R were treated with 1 µM NRF2 inhibitor (ML385; Sigma-Aldrich) [42] at the time of reoxygenation, with or without the addition of EV-H5 (2.5 × 10e4 EV particles/cell). Subsequently, the cells were analyzed using fluorescence microscopy and qPCR methods at 4 and 24 h post-reoxygenation. Cells that were subjected to OGD/R without any additional treatment served as controls.

### Immunocytofluorescence

Cells were seeded on Geltrex-coated glass bottom 24-well plates (Ibidi) at a density of 2 × 10e4 cells/cm^2^ and cultured under the conditions described above. The next day, the cells were exposed to OGD/R insult with or without EV-H5 treatment or NRF2 inhibition. After 4 or 24 h, cells were fixed in 3.7% formaldehyde in PBS and permeabilized with 0.1% Triton X-100 in PBS. Non-specific antibody binding sites were blocked with 2% FBS solution in PBS. Samples were incubated with primary antibody against NRF2 overnight at 4 °C. After rinsing with PBS, secondary antibody conjugated to Alexa Fluor 546 was added and incubated for 45 min at RT. Antibody specifications are listed in Table 2. Samples were counterstained with DAPI (4’,6-diamidino-2-phenylindole, dihydrochloride; Molecular Probes/Thermo Fisher Scientific) for DNA visualization. All images were acquired using a Leica DMI6000B fluorescence microscope with LasX software (Leica Microsystems GmbH, Wetzlar, Germany) and were captured with identical binning, excitation and exposure settings. The percentage of cells with nuclear localization of NRF2 was reported for all tested conditions. Quantification of fluorescence in the collected images was performed using FiJi ImageJ freeware (NIH, Bethesda, MD, USA) and presented as the average intensity of NRF2 fluorescence from the nuclear area relative to the total fluorescence signal of whole cells. Fluorimetry was expressed as relative fluorescence units (RFU). Visualization of NRF2 in CMs after ML385 treatment was additionally performed using a confocal microscope (Zeiss LSM 900 with Airyscan 2).

**Table 2:** List of antibodies used in the study

Antibody	Specification			Dilution
**Primary antibodies**				
anti-NRF2	polyclonal rabbit IgG, Invitrogen #PA5-27882		1:100	
anti-Syntenin	polyclonal goat, IgG, Invitrogen #PA5-18595		1:500	
anti-Flotillin1	polyclonal goat, IgG, Invitrogen #PA5-1853		1:1000	
anti-CD81	monoclonal mouse, IgG1, kappa, Invitrogen #MA5-13548		1:500	
anti-OCT4	monoclonal mouse, IgG, Invitrogen #MA1-104		1:1000	
anti-β-actin	monoclonal mouse, IgG2b, Invitrogen #MA5-15739		1:2000	
anti-β-tubulin	monoclonal mouse, IgG2a, Invitrogen #MA5-16308		1:2000	
anti-PTEN	monoclonal rabbit, IgG, Cell Signaling Technology #9188		1:1000	
anti-pAkt (Ser473)	monoclonal rabbit, IgG, Cell Signaling Technology #4060		1:2000	
anti-Akt (pan)	monoclonal rabbit, IgG, Cell Signaling Technology #4991		1:1000	
anti-pErk1/2 (Thr202/Tyr204)	monoclonal rabbit, IgG, Cell Signaling Technology #4370		1:2000	
anti-Erk1/2	monoclonal rabbit, IgG, Cell Signaling Technology #4695		1:1000	
anti-pAMPK (Thr172)	monoclonal rabbit, IgG, Cell Signaling Technology #5831		1:1000	
anti-αAMPK	monoclonal rabbit, IgG, Cell Signaling Technology #4991		1:1000	
anti-GAPDH	monoclonal mouse, IgG1, Invitrogen #MA5-15738		1:2000	
anti-Vinculin	monoclonal mouse, Sigma-Aldrich #V9264		1:1000	
anti-PRDX6	monoclonal mouse, IgG1, Millipore #MABN1797		1:1000	
anti-GSTP1	monoclonal mouse, IgG1, Cell Signaling Techn. #3369		1:1000	
anti-HSP90B	monoclonal rabbit, IgG, ABclonal #A19574		1:1000	
anti-PRDX1	polyclonal rabbit, IgG, ABclonal #A22008		1:1000	
anti-CYPB	polyclonal rabbit, IgG, GeneTex #GTX118372		1:1000	
anti-HIF1α	polyclonal rabbit, IgG, GeneTex #GTX127309		1:1000	
anti-hydroxy-HIF1α	monoclonal rabbit, IgG, Cell Signaling Techn. #3434		1:1000	
anti-HIF2α	monoclonal rabbit, IgG, Bethyl Laboratories #A700-003-T		1:1000	
**Secondary antibodies**				
anti-rabbit, AF 546	goat IgG, Invitrogen #A-11,035		IF (1:500)	
anti-mouse, HRP	goat IgG, Cell Signaling Technology		WB (1:2000)	
anti-rabbit, HRP	goat IgG, Cell Signaling Technology		WB (1:2000–6000)	
anti-goat, HRP	rabbit IgG, Invitrogen #R-21,459		WB (1:2000)	

### Immunoblotting

Cell lysates were prepared in RIPA buffer (Sigma) supplemented with Halt Protease and Phosphatase Inhibitor Cocktail (Thermo Scientific) and sonicated three times for 10 s at 30 s intervals. EVs were lysed with 5:1 RIPA buffer. Lysates were centrifuged at 4°C for 15 min at 10,000 × g to isolate the protein fraction. Protein concentration was determined by BCA assay (Thermo Fisher Scientific). Protein samples were mixed with 4x Laemmli sample buffer (Bio-Rad) and boiled at 95⁰C for 5 min. Then 25 µg or 4 µg of cell or EV lysate, respectively, were separated by electrophoresis on 4–12% SDS-polyacrylamide gels. Proteins were then transferred to polyvinylidene difluoride (PVDF) membrane (Bio-Rad) using a semi-dry transfer at 25 V, 1.3 A for 7–10 min (depending on protein size) in the Trans-Blot Turbo RTA Mini PVDF Transfer Kit (Bio-Rad). After blocking non-specific antibody binding sites with SuperBlock T20 (TBS) blocking buffer (Thermo Fisher Scientific), the membranes were incubated overnight at 4°C with primary antibodies diluted in 3% BSA solution in TBST. After washing, the membranes were incubated with horseradish peroxidase (HRP)-conjugated secondary antibodies. The list of antibodies is provided in Table 2. Protein detection was performed using the Luminata Crescendo Western HRP Substrate (Merck) and a ChemiDoc XRS + chemiluminescence imaging system (Bio-Rad). If necessary, membranes were stripped using the Restore PLUS Western Blot Stripping Buffer (Thermo Scientific) and reprobed with a different set of antibodies (at a different molecular weight). Densitometric analysis was performed using the Quantity One software (Bio-Rad). The results are presented as the ratio of pixel density of phosphorylated proteins to total proteins relative to the control protein (either GAPDH, β-actin, β-tubulin, vinculin or CYPB).

### Contractility and calcium signaling in CMs

Functional analysis of CMs was performed using combined atomic force and fluorescence microscopy (Bioscope Catalyst AFM mounted on a Zeiss inverted optical microscope). Prior to analysis, Fluo-4 probe was added to the cells and incubated for 30 min. When the cells were ready for analysis, the Petri dish containing CMs was placed on the AFM stage and the probe was aligned in the center of the optical path. This allowed rapid selection of cells for analysis using the optical microscope view. 10 cells were selected for each condition. An MLCT-bio probe with a nominal spring constant of 0.01 N/m was selected for analysis. During the analysis, the cells were kept at 37°C. Briefly, once a cell was selected for analysis, the AFM was activated to make contact with the cell. A custom-made script allowed simultaneous triggering of both AFM and fluorescence imaging. Fluorescence images were acquired as a time series with the exposure time set to obtain a few frames per second. The camera settings were exactly the same for all cells analyzed. Fluorescence detection was performed with an ORCA-Flash4.0 LT3 digital CMOS camera using a ×20 air objective with a numerical aperture of 0.3 NA. The data obtained were analyzed using custom-made software. Briefly, the raw data obtained from the AFM analysis were converted into force values using the calibration file. Calibration was performed before and after analysis and the slope value of the curves was averaged. The curves obtained were used to determine both frequency and contraction force. A detailed description of force spectroscopy measurement using AFM can be found elsewhere [43, 44]. In the case of fluorescence data, calcium fluxes were determined using a dedicated plugin for ImageJ software.

### Statistical analysis

Data are presented as mean with standard deviation (SD). Individual points of the measurements are shown in each graph. Statistical analysis was performed using GraphPad Prism 8.4.0 software. First, the Shapiro-Wilk test was used to assess the normal distribution of the data. Next, homogeneity of variances was analyzed by F-test (two groups) or Brown-Forsythe test (three or more groups). Statistical significance was measured by paired standard Student’s t-test when comparing two groups with equal variances, or by one-way ANOVA for multiple comparisons. Post hoc tests included Tukey’s test for normal distribution of data and equal variances, or Welch ANOVA with Dunnett’s test for unequal variances. When the data were not normally distributed, the nonparametric Kruskal-Wallis test with Dunn’s post hoc test was used. Differences at *p* < 0.05 were considered statistically significant. Statistical tests used for data analysis are indicated in each figure legend and individual *p*-values are given in each graph.

## Results

### hiPS-EVs derived from different oxygen conditions exhibit a distinct proteomic profile

**Fig. 1:**
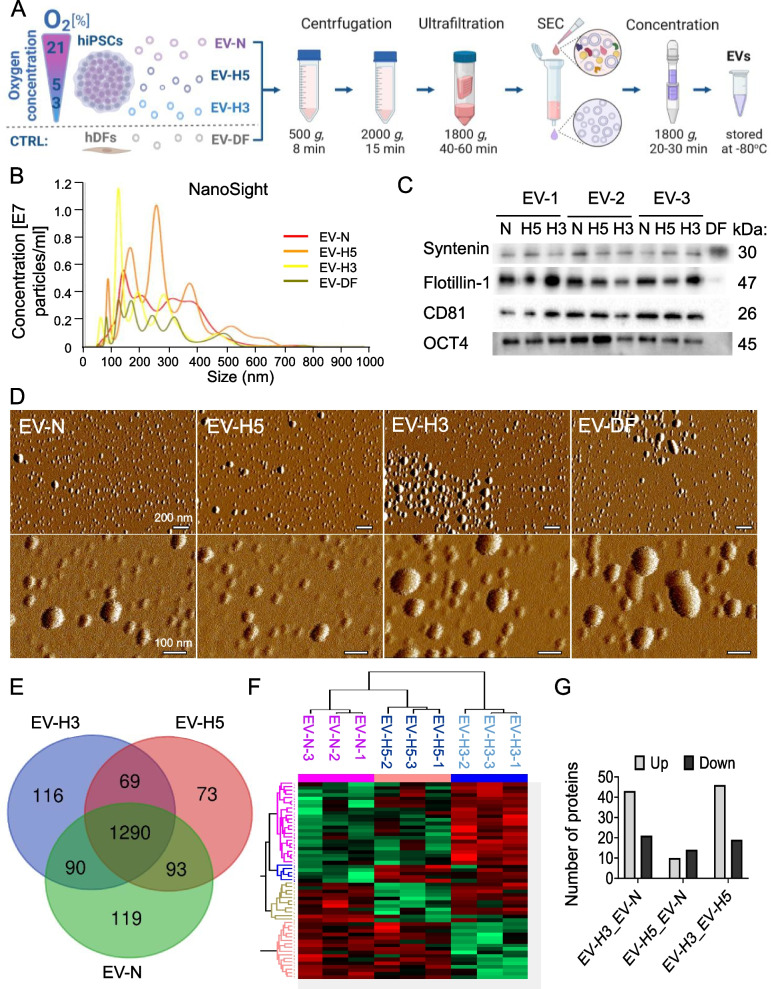
Characterization of the extracellular vesicles (EVs) used in the study. **A **Schematic representation of the EV isolation procedure. **B** Representative histograms of EV size and concentration measured with the NanoSight instrument. **C** Western blot of proteins typical of EVs (syntenin, flotillin1, CD81) and a marker of pluripotency (OCT4). **D** Atomic force microscopy images of EVs. Scale bar 200 nm (left panel) or 100 nm (right panel). **E** Venn diagram showing common and distinct proteins identified in hiPS-EVs derived from various oxygen conditions: normoxia (21% O_2_; EV-N) and hypoxia at 5% O_2_ (EV-H5) and hypoxia at 3% O_2_ (EV-H3). Only proteins that were present in all three samples within a group, based on at least two peptides and detected in at least 6 of the total 9 samples were included. **F** Heatmap of differentially expressed proteins in hiPS-EVs derived from different oxygen conditions based on mass spectrometry analysis. **G** Quantification of differentially expressed proteins in hiPS-EVs (inclusion criteria: proteins in all 3 samples based on at least 2 peptides)

We used three hiPSC lines to investigate the overall effect of oxygen concentration on the properties of hiPS-EVs during cell culture, to avoid any bias caused by individual cell lines. Each cell line was cultured in a serum-free medium, under three different oxygen concentrations: (i) 3% physiological hypoxia, (ii) 5% O_2_, and (iii) 21% O_2_ atmospheric oxygen concentration. We have confirmed the presence of hypoxia-inducible factors (HIFs) in these cells by Western blot. The activity of HIF1α was predominantly detected at hypoxia 3% O_2_, whereas HIF2α was significantly upregulated under both hypoxic conditions, compared to normoxia (Additional File 1: Fig. S1). We then collected conditioned media from these cells and used them to isolate EVs *via* UF + SEC methodology (Fig. 1A) [22]. We also utilized EVs from therapeutically inert human dermal fibroblasts (hDFs; EV-DF) for comparison. To comply with the guidelines of the International Society for Extracellular Vesicles (ISEV) (MISEV 2018) [6], we characterized the EVs by NTA, Western blot, and AFM methods (Fig. 1A-C). Detailed characteristics, including EV particle yield and protein content, can be found elsewhere [22]. As evaluated using the NanoSight instrument, the EVs had a medium size of about 220 nm in diameter. It is noteworthy that EVs produced under hypoxic conditions and EV-DF were smaller in size compared to those produced under normal conditions (with a mean size of 200 nm for EV-H3 and 180 nm for DF-EVs) (Fig. 1B). The yield of EV particles was highest for H5 condition (4.3 ± 2 × 10e9/ml of conditioned medium), in comparison to other conditions (2.9 ± 1.7 and 1.6 ± 0.6 × 10e9/ml of conditioned medium for N and H3, respectively) [22], although the difference was not statistically significant. Additionally, EVs derived from all cell types expressed typical protein markers, such as syntenin, flotillin 1, and CD81 (Fig. 1C, Additional File 2: Fig. S2). Furthermore, hiPS-EVs contained the pluripotency marker OCT4, which is characteristic of their parental cells. All EVs showed a typical circular shape (Fig. 1D).

Next, we aimed to describe the molecular profile of hiPS-EV samples harvested at different oxygen concentrations. Previously, we determined miR-302b-3p as the predominant miRNA in hiPS-EVs [22], however, based on miRNA-target gene interactions, it was not directly linked to anti-oxidative function. Therefore, we hypothesized that the protein cargo may have significant implications for cardio-protection. Therefore, we performed proteomic analysis of hiPS-EV from different oxygen conditions.

The complete data of the proteomic analysis are available at 10.57903/UJ/VZDNQW. A total of 2615, 2408, and 2566 non-redundant proteins were identified based on at least two peptides in EVs derived from normoxia, hypoxia at 5% O_2_, and hypoxia at 3% O_2_, respectively (Additional File 3: Fig. S3A). From this pool, 1592, 1525, and 1565 proteins were common to all three samples within the groups: N, H5, and H3, respectively (Additional File 3: Fig. S3B). Further comparison revealed that 1290 proteins were common to EVs derived from all three different oxygen concentrations (detected in at least 6 out of 9 samples) (Fig. 1E). Enriched pathways for these proteins indicated cellular processes related to metabolism of RNA and proteins, cellular response to stress and stimuli, developmental biology, mitosis, vesicle-mediated transport and others (Additional File 4: Fig. S4). Next, we analyzed proteins specific for EVs derived from each oxygen condition. Enriched pathways for EV-N (119 proteins) included: cellular component organization or biogenesis, organelle localization and intracellular transport (Additional File 5: Fig. S5A). In turn, proteins typical for EV-H5 (73 proteins) pinpointed regulation of protein metabolic process, regulation of protein kinase activity, phosphorylation and protein modification, among others (Additional File 5: Fig. S5B). On the contrary, pathways enriched for proteins detected typically in EV-H3 (116 proteins) suggested RNA metabolic process, cellular nitrogen compound metabolic process, non-coding RNA processing (Additional File 5: Fig. S5C). To further investigate the molecular footprint of protein composition between EVs harvested under different oxygen conditions, we performed differential protein analysis of the common proteins. The differentially expressed proteins were outlined in a heat map forming 4 distinct clusters (Fig. 1F). It is noteworthy that EV-H5 showed more similarities to EV-N than to EV-H3. The comparison between EV-H5 and EV-H3 revealed the highest number of differentially expressed proteins, with 46 and 19 upregulated or downregulated proteins, respectively (Fig. 1G). The subsequent comparison between EV-H3 and EV-N resulted in 43 and 21 upregulated or downregulated proteins, respectively. In contrast, the comparison of EV-H5 and EV-N returned only 10 and 14 upregulated or downregulated proteins, respectively (Fig. 1G).

**Fig. 2:**
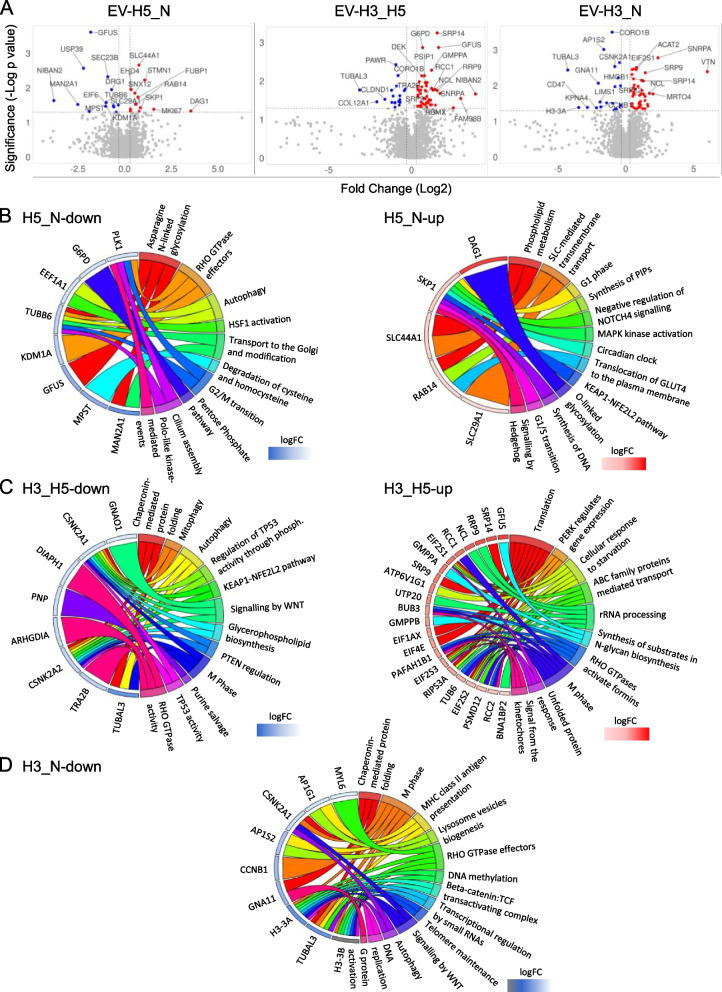
Differential expression analysis of proteins detected in hiPS-EVs derived from different oxygen conditions by liquid chromatography-mass spectrometry (LC-MS/MS). **A** Volcano plots of differentially expressed proteins compared in pairs. Gene set enrichment analysis of differentially expressed proteins in each pair of EVs compared (**B** EV-H5 vs. EV-N; **C** EV-H3 vs. EV-H5; **D** EV-H3 vs. EV-N), visualized in chord plots using the GOplot package in R

Proteins that were enriched differently across distinct EV types are indicated in volcano plots (Fig. 2A). Next, they were subjected to pathway enrichment analysis using the ReactomePA package in R. Pathways that were downregulated in EV-H5, in comparison to EV-N, comprise N-linked glycosylation, RHO GTPase effectors, autophagy, cell cycle regulation, and the pentose phosphate pathway (Fig. 2B, left). In contrast, the proteins that were upregulated suggest a possible impact on cell survival through phospholipid metabolism, transmembrane transport, MAPK kinase activation and the KEAP1-NFE2L2 pathway (Fig. 2B, right). Notably, the KEAP1-NFE2L2 pathway was downregulated in EV-H3 as compared to EV-H5, together with chaperonin-mediated protein folding, mitophagy, and TP53 regulation through phosphorylation (Fig. 2C, left). In contrast, EV-H3 was found to enrich for proteins that regulate translation, respond to cellular starvation and process rRNA compared to EV-H5 (Fig. 2C, right). Further analysis of proteins enriched under the most contrasting oxygen conditions (EV-H3 versus EV-N) suggested a reduction in chaperonin-mediated protein folding, M-phase, DNA methylation, autophagy, DNA replication, RHO GTPase effectors, and WNT signaling, among others, compared to EV-N (Fig. 2D, left). Conversely, no enriched pathways were detected in the analysis of upregulated proteins within this contrast. Thus, we performed an additional analysis of proteins differentially expressed between EV-H3 and EV-N using the Ingenuity software. The obtained results indicated activated HIF1α signaling, increased glycolysis and enhanced cell proliferation (Additional File 6: Fig. S6).

### hiPS-EVs reduce oxidative stress and inhibit inflammation in CMs after OGD/R

**Fig. 3:**
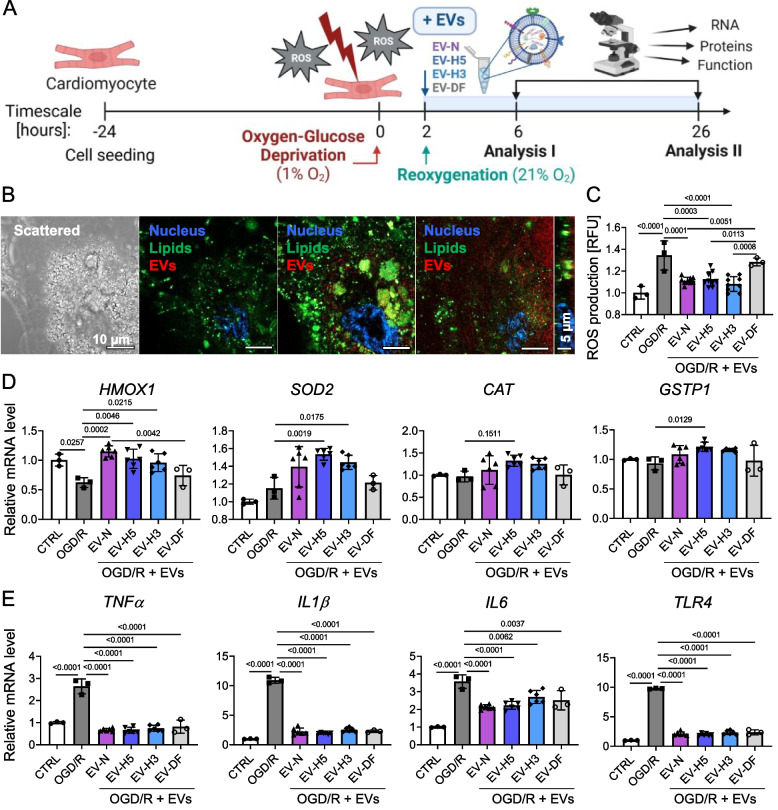
Comparison of the cytoprotective effect of hiPS-EVs derived from three hiPSC lines cultured under different oxygen conditions (21, 5 and 3% O_2_, designated EV-N, EV-H5 and EV-H3, respectively) and EVs derived from dermal fibroblasts (EV-DF) on cardiomyocytes (CMs) subjected to oxygen-glucose deprivation followed by reoxygenation (OGD/R). CMs were derived from hiPSCc (L-3). Analyses were performed 4 h after reoxygenation. **A **Schematic representation of the experimental pipeline. **B **Confocal microscopy images of DiD-labelled EVs (red) taken up by CMs stained green with DiO. Representative images of different cell depths are shown. The scale bar is 10 μm or 5 μm in cross section. **C** Measurement of reactive oxygen species (ROS) levels in CMs treated or untreated with hiPS-EVs or EV-DF. Cells not exposed to OGD/R served as control (CTRL). Data are expressed as relative fluorescence units (RFU), *n* = 3–9. Measurement of the expression level of antioxidant genes (*HMOX1, SOD2, CAT, GSTP1*) (**D**) or genes encoding pro-inflammatory factors (*TNFα, IL1β, IL6, TLR4*) (**E**) by real-time qPCR. *GAPDH* and *B2M* were used as endogenous controls, *n* = 3–6. All data are presented as mean ± SD. Statistical significance was tested using one-way ANOVA followed by Tukey’s post-hoc test. Significant *p*-values (*p* < 0.05) are shown in each graph

To verify the biological effect of hiPS-EVs derived from different oxygen conditions on CMs, we used an in vitro model of OGD/R (Fig. 3A), which mimics the natural process of CM injury in the post-infarcted heart. We used CMs generated from hiPSCs (L3) in this assay. Following the differentiation protocol, we obtained functional CMs exhibiting contractile activity in cell culture (Additional File 7). We confirmed the expression of cardiac markers (*GATA4* and *TNNT2*) in these cells (Additional File 8: Fig. S7A), which was accompanied by a significant decrease in the expression of pluripotency markers (*OCT4, NANOG*) (Additional File 8: Fig. S7B). In the OGD/R model, CMs were exposed to severe hypoxia (1% O_2_) for 2 h in the environment of an acidic buffer deprived of glucose. Immediately thereafter, to simulate reperfusion, the cells were transferred to standard cell culture medium at atmospheric oxygen concentration with the simultaneous addition of EVs. Untreated cells were used as a control. As there is a very narrow time window for cardioprotective interventions in the heart after reperfusion [44, 45], we analyzed the functionality of CMs and their molecular signature at 4 and 24 h after reoxygenation using various assays.

First, we demonstrated that CMs are able to internalize hiPS-EVs (Fig. 3B), which is essential for the biological activity of the EV cargo. CMs were then used in the OGD/R assay. ROS levels were assessed 4 h after reoxygenation. Rapid changes in oxygen levels resulted in increased ROS production (1.34 ± 0.13 vs. 1 ± 0.06 in control), which was significantly reduced upon hiPS-EVs treatment (1.11 ± 0.03 for EV-N, 1.12 ± 0.07 for EV-H5 and 1.08 ± 0.07 for EV-H3), in contrast to EV-DF (1.28 ± 0.04) (Fig. 3C). Simultaneously, we measured transcript levels of genes involved in antioxidant and detoxification response, including *HMOX1, SOD2, CAT* and glutathione S-transferase pi 1 (*GSTP1*). Accordingly, ROS insult resulted in a significant downregulation of *HMOX1* in CMs, whose expression level was restored after treatment with all types of hiPS-EVs (Fig. 3D). Transcription of *SOD2* was significantly upregulated after addition of EV-H5 and EV-N, whereas mRNA levels for *GSTP1* and *CAT* (although not significant for the latter) increased only after treatment with EV-H5 (Fig. 3D).

Given that first stages of heart remodeling after infarction are associated with the release of pro-inflammatory cytokines [4], we measured the mRNA levels of genes encoding tumor necrosis factor alpha (*TNFα*), interleukin 6 (*IL6*), interleukin 1 beta (*IL1β*) and toll-like receptor 4 (*TLR4*). After OGD/R, we observed a significant increase in the expression level of all genes analyzed, which was significantly reduced after treatment with hiPS-EVs from all oxygen conditions as well as after the addition of EV-DF (Fig. 3E).

### EV-H5 trigger pro-survival pathways in CMs more effectively than EV-H3 or EV-N

**Fig. 4:**
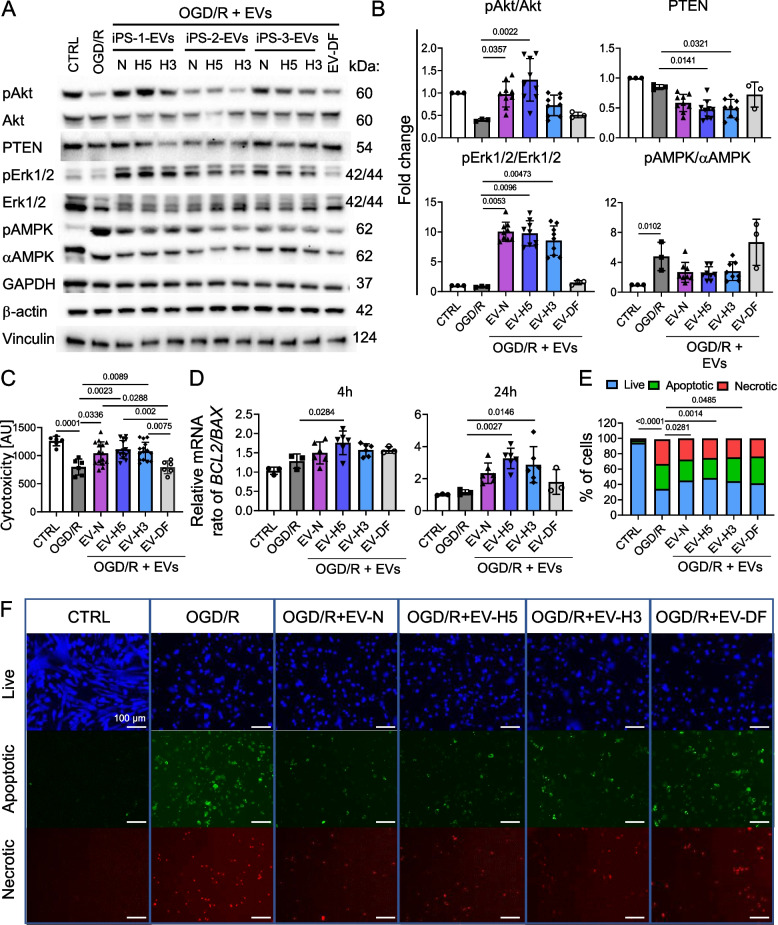
Effects of hiPS-EVs derived from three hiPSC lines cultured at different oxygen concentrations (21, 5 and 3% O_2_, designated EV-N, EV-H5 and EV-H3, respectively) and dermal fibroblast-derived EVs (EV-DF) on pro-survival pathways and apoptosis in cardiomyocytes (CMs) subjected to oxygen-glucose deprivation followed by reoxygenation (OGD/R). CMs were derived from hiPSCc (L-3). Cells not treated with OGD/R were used as control (CTRL). **A **Western blot detection of activated kinases: Akt (Ser473), Erk1/2 (Thr202/Tyr204), AMPK (Thr172) and their total protein levels, as well as the total level of PTEN, 24 h after OGD/R. GAPDH, β-actin and vinculin were used as controls. Representative membranes are shown. **B **Densitometric analysis of protein levels detected by Western blot, relative to control, *n* = 3–9. (**C**) Analysis of cytotoxicity in CMs 24 h after OGD/R using Cell Counting Kit-8. Data are presented in arbitrary units (AU), *n* = 6–15. (**D**) Ratio of mRNA levels of anti- and pro-apoptotic genes (*BCL2* and *BAX*, respectively) measured in CMs at 4 (left) and 24 h (right) after reoxygenation by real-time qPCR. *GAPDH* and *B2M* were used as endogenous controls, *n* = 3–6. Analysis of apoptosis of CMs at 24 h post-reoxygenation (**E**, **F**). Percentage of live, apoptotic and necrotic cells in each experimental group (**E**), *n* = 4–6 and corresponding fluorescence microscopy images (**F**). Scale bar is 100 μm. All data are presented as mean ± SD. Statistical significance was tested using the Kruskal-Wallis test with Dunn’s post-hoc test (**B**), one-way ANOVA with Tukey’s post-hoc test (**C**) or Dunnett’s post-hoc test (D) or two-way ANOVA with Tukey’s test (**E**). Significant *p*-values (*p* < 0.05) are shown in each graph

To further investigate the cytoprotective function of hiPS-EVs derived from different oxygen conditions on CMs, we performed a detailed analysis of the activities of pro-survival proteins and their interactomes after OGD/R. We focused on the phosphatase and tensin homolog (PTEN)/Akt axis, Erk1/2 kinases and the master sensor kinase of metabolic stress - adenosine monophosphate-activated protein kinase (AMPK). At 4 h post-reperfusion, we detected increased levels of phosphorylation of Akt, Erk1/2 and AMPK and decreased levels of PTEN, a negative regulator of Akt (Additional File 9: Fig. S8, Additional File 10: Fig. S9), indicating activation of endogenous repair mechanisms in damaged CMs. This activation was more pronounced after treatment with hiPS-EVs, in particular EV-H5. In contrast, the level of Akt phosphorylation decreased in CMs at 24 h post-OGD/R (to the value of 0.44 ± 0.1 relative to control set to 1) and significantly increased in cells treated with EV-H5 (reaching mean value of 1.29 ± 0.4, in comparison to 0.97 ± 0.3, 0.72 ± 0.2 and 0.49 ± 0.06 for EV-N, EV-H3 and EV-DF) (Fig. 4A, B; Additional File 11: Fig. S10). The upregulation of Akt in CMs after hiPS-EVs was accompanied by a decrease in the level of PTEN, whose expression level was significantly reduced in samples treated with EV-H5 and EV-H3 (respective values: 0.4 ± 0.1 and 0.41 ± 0.2, in comparison to 0.85 ± 0.04 for the OGD/R sample). Impressively, the phosphorylation level of Erk1/2 substantially increased in CMs treated with all types of hiPS-EVs, in comparison to the OGD/R group (relative protein levels: 10.1 ± 1.7, 9.7 ± 2.2, 8.1 ± 2.3 and 0.8 ± 0.1 for EV-N, EV-H5, EV-H3 and OGD/R) (Fig. 4A, B; Additional File 11: Fig. S10). In contrast, DF-EVs had little effect on the phosphorylation of Erk1/2 (1.5 ± 0.3). With respect to AMPK, stress conditions of OGD/R induced its phosphorylation at 24 h post-reoxygenation (to the level of 4.8 ± 2.3, compared to 1 in control) (Fig. 4A, B). The addition of hiPS-EVs reduced the level of AMPK phosphorylation by almost 50% (respective values: 2.7 ± 1.3, 2.5 ± 0.9, 2.8 ± 1.3 for EV-N, EV-H5 and EV-H3). At the same time, EV-DF had no effect on AMPK activity (6.7 ± 3.1), which remained considerably higher than after treatment with all types of hiPS-EVs.

The induction of oxidative stress was accompanied by a substantial decrease in the metabolic activity of CMs, as measured by the Cell Counting Kit-8, 24 h after OGD/R (by 33%; relative values: 827 ± 151arbitrary units; a.u. in OGD/R group vs. 1239 ± 119 a.u. in control) (Fig. 4C). Treatment with hiPS-EVs increased cellular metabolism, in particular EV-H5 (1112 ± 157 a.u.), which was also significantly higher than after the use of EV-DF (801 ± 126 a.u.).

Knowing that dysregulation of ROS homeostasis leads to CM damage and death, we measured the rate of apoptosis in CMs after OGD/R. First, we examined changes in the transcript level of pro- and anti-apoptotic genes *BAX* and *BCL2*, since the decrease in the BCL2/BAX ratio directs the cell towards apoptosis. At 4 h post reoxygenation, the level of *BCL2*/*BAX* increased from the value of 1 ± 0.1 in control CMs to 1.8 ± 0.3 in CMs treated with EV-H5, in comparison to 1.3 ± 0.2 in cells from the OGD/R group (Fig. 4D, left). The effect of EV-H5 was even more pronounced 24 h post treatment (respective ratios of transcript levels: 1 ± 0.04, 3.3 ± 0.6 and 1.15 ± 0.2 for control, EV-H5 and OGD/R samples) (Fig. 4D, right).

Furthermore, we measured the percentage of live, apoptotic (Annexin V positive) and necrotic cells in CMs untreated or treated with hiPS-EVs at 24 h post reoxygenation. The highest percentage of live cells was observed in the control group (94.2 ± 5.4%), which was substantially reduced in the OGD/R condition (34.1 ± 7.2%). Treatment with hiPS-EVs improved cell survival by 10–11% for EV-H3 and EV-N (reaching values of 44.3 ± 7.6% and 45 ± 5.7%, respectively) and most significantly for EV-H5 (48.3 ± 8.7%). In contrast, EV-DF improved cell survival at a moderate level (41.2 ± 8.7%) (Fig. 4E, F).

Given the highest activation of pro-survival Akt kinase, accompanied by increased transcription of anti-apoptotic and anti-oxidative genes in CMs, and the highest cell survival upon treatment with EV-H5, we selected these EVs for further studies.

### EV-H5 restore calcium signaling and contractility in CMs after OGD/R

**Fig. 5:**
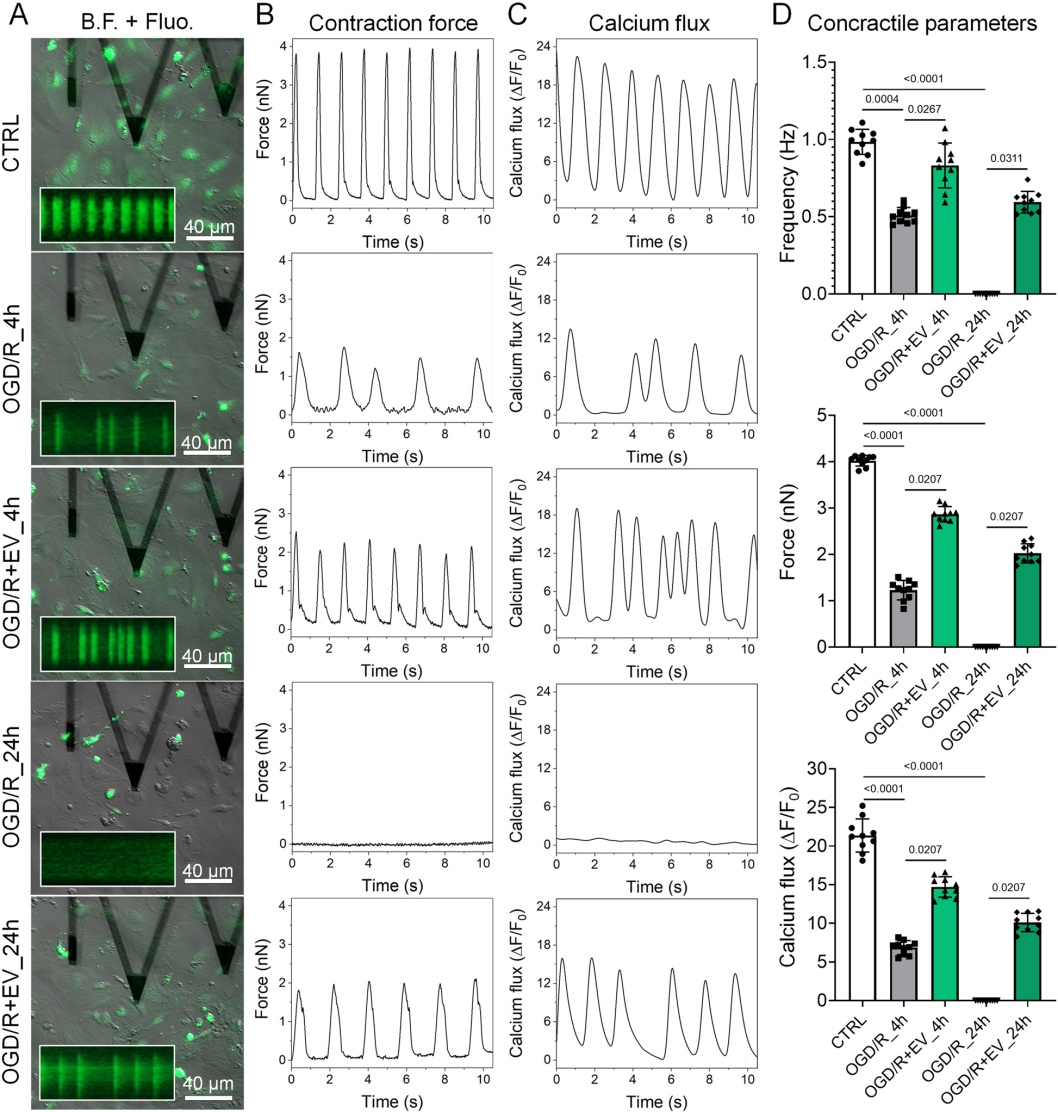
Functional analysis of contractility and calcium handling in CMs subjected to oxygen-glucose deprivation followed by reoxygenation (OGD/R) and treated with hiPS-EVs derived from 5% oxygen (EV-H5, L-3) using combined atomic force (AFM) and fluorescence microscopy. **A** Bright field (B.F.) and fluorescence (Fluo.) micrographs of CMs during analysis. Insets show example calcium traces of cells measured simultaneously with the AFM probe (visible as large triangles in the images). **B** Plots of cell contraction force determined by AFM followed by calcium handling plots (**C**). **D** Quantitative analysis of the functional parameters obtained: frequency and force of contractions and calcium flux, *n* = 10. Data are presented as mean ± SD. Statistical significance was tested using the Kruskal-Wallis test with Dunn’s post-hoc test. Significant *p*-values (*p* < 0.05) are shown in each graph

Knowing that calcium signaling plays a central role in excitation-contraction coupling in CMs [46], we performed a direct measurement of contractile force and calcium flux in CMs challenged with OGD/R. For this purpose, we used an advanced technology based on AFM. CMs treated or untreated with EV-H5 were analyzed 4 and 24 h after reoxygenation. Representative images of cells with the AFM probe are shown in Fig. 5 (left panel). In control cells, the contraction frequency was 0.98 ± 0.08 Hz, the contraction force was 4.02 ± 0.11 nN, and the calcium level was 21.37 ± 2.14 a.u. (Fig. 5B-D). In contrast, cells exposed to OGD/R showed significantly altered contractile parameters and even stopped beating 24 h after reoxygenation. At 4 h after OGD/R, the cells showed a contraction frequency of 0.51 ± 0.05 Hz, a contraction force of 1.23 ± 0.21 nN and a calcium concentration of 6.86 ± 0.89 a.u. Importantly, despite these significant changes, the cells were still alive. However, when CMs were treated with EV-H5, they maintained their contractility even 24 h after reoxygenation. A significant improvement in contractile parameters was readily detected at 4 h after reoxygenation, when the cells exhibited a contraction frequency of 0.83 ± 0.14 Hz, a contraction force of 2.87 ± 0.16 nN, and a calcium level of 14.71 ± 1.31 a.u. At 24 h after OGD/R, CMs untreated with EVs showed no signs of normal contractile activity (all parameters at 0). On the other hand, cells treated with EV-H5 showed a contraction frequency of 0.59 ± 0.07 Hz, a contraction force of 2.02 ± 0.21 nN and a calcium flux of 10.11 ± 1.18 a.u. The results obtained indicate that EV-H5 are able to maintain the contractility of CMs after OGD/R.

### EV-H5 are enriched in antioxidant proteins

**Fig. 6:**
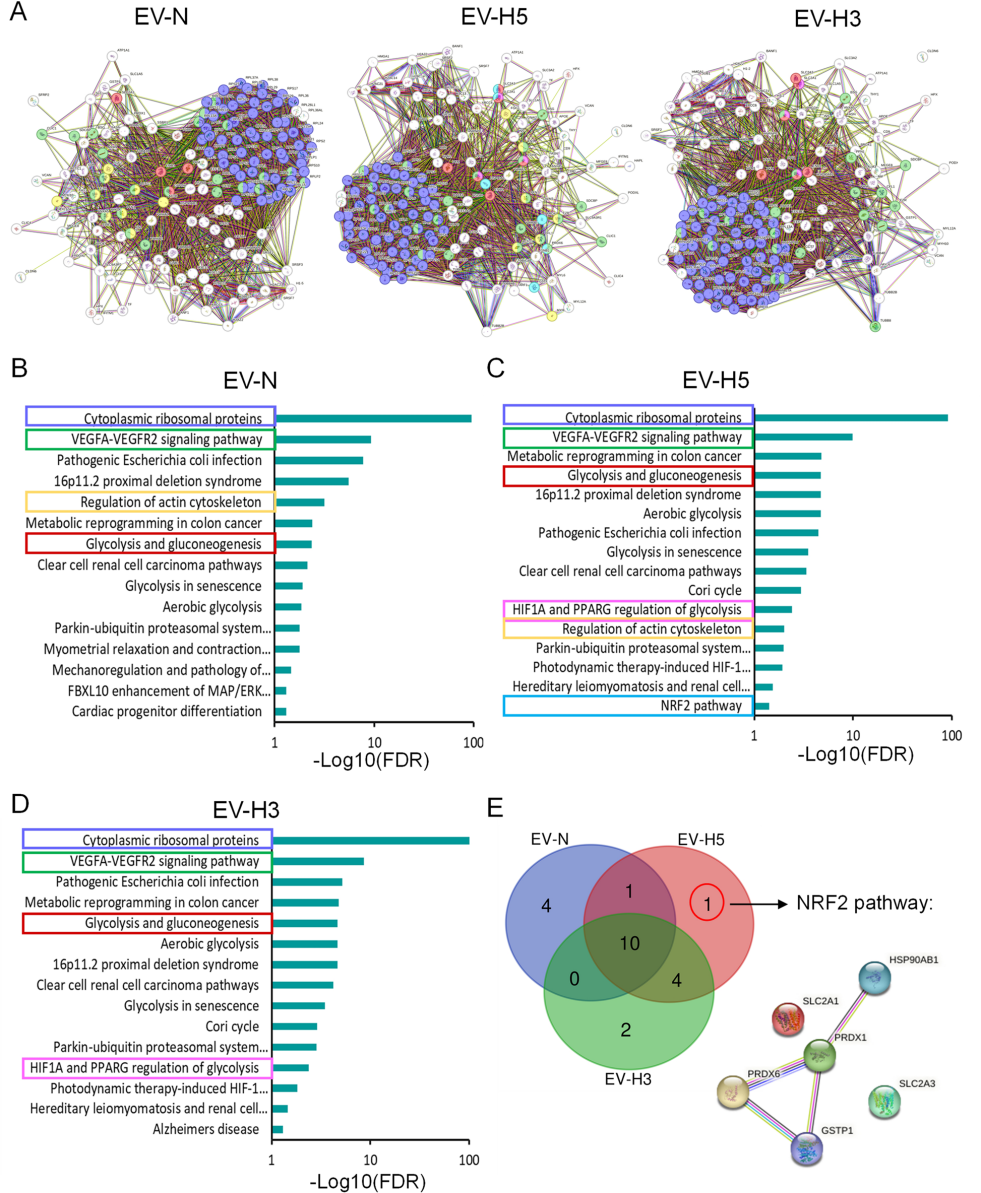
Identification of the most abundant proteins with cytoprotective and antioxidant function in hiPS-EVs based on LC-MS/MS analysis. The 5% most abundant proteins in hiPS-EVs derived from three hiPSC lines cultured under different oxygen conditions (normoxia − 21% O_2_ - EV-N; hypoxia 5% O_2_ - EV-H5; hypoxia 3% O_2_ - EV-H3) were determined by intensity based absolute quantitation (iBAQ) analysis. **A** Protein-protein interaction (PPI) networks of the identified proteins generated in the STRING database v12 with color-coding of selected proteins matching the pathways in panels B-D. **B** Functional enrichment analysis of the top 5% proteins in EV-N (**B**), EV-H5 (**C**) and EV-H3 (**D**) using the STRING web tool based on WIKIPathways. **E** Venn diagram showing the relationship between the pathways identified in hiPS-EVs in each oxygen condition tested and the PPI network of proteins from the NRF2 pathway

Although differential protein expression analysis revealed substantial differences in the pathways enriched under specific oxygen conditions, it was still unknown whether these proteins were abundant enough to exert their molecular function in recipient cells. In our previous study, we screened the miRNA content of hiPS-EVs and identified miR-302b-3p as the most abundant miRNA in hiPS-EVs, with the highest prevalence in EV-H5 [22]. Importantly, one of the target genes of this miRNA is PTEN, whose expression level was significantly downregulated in CMs subjected to OGD/R and treated with EV-H5. In this work, we went one step further and analyzed the most abundant proteins in hiPS-EVs. For this purpose, we performed intensity-based absolute quantification (iBAQ) analysis of the most abundant proteins (top 5%) in EVs derived from different oxygen conditions. Protein-protein interaction (PPI) network analysis confirmed a higher degree of similarity between EV-H5 and EV-N, in contrast to EV-H3 (Fig. 6A). Pathway enrichment analysis using WIKIPathways revealed a similar set of pathways in EVs derived from different oxygen concentrations (Fig. 6B-D). The most prominent common terms pointed to cytoplasmic ribosomal proteins, the VEGFA-VEGFR2 signaling pathway, which may play a role in supporting angiogenesis, and glycolysis and gluconeogenesis. Common pathways for EV-N and EV-H5 also included regulation of the actin cytoskeleton, which may be important for maintaining sarcomere architecture in CMs. Strikingly, the most abundant proteins in EV-H5 differed from other EV types in one pathway, the antioxidant NRF2 pathway (Fig. 6C, E). Proteins from this pathway included: heat shock protein 90 alpha family class B member 1 (HSP90AB1, also known as HSP90B or HSP84), PRDX1, PRDX6, GSTP1, solute carrier family 2 member 1 and 3 (SLC2A1, SLC2A3). The PPI network showed a close interaction between HSP90B, PRDX1, PRDX6 and GSTP1, which were selected for further investigation.

### EV-H5 stimulate antioxidant response via NRF2 signaling

**Fig. 7:**
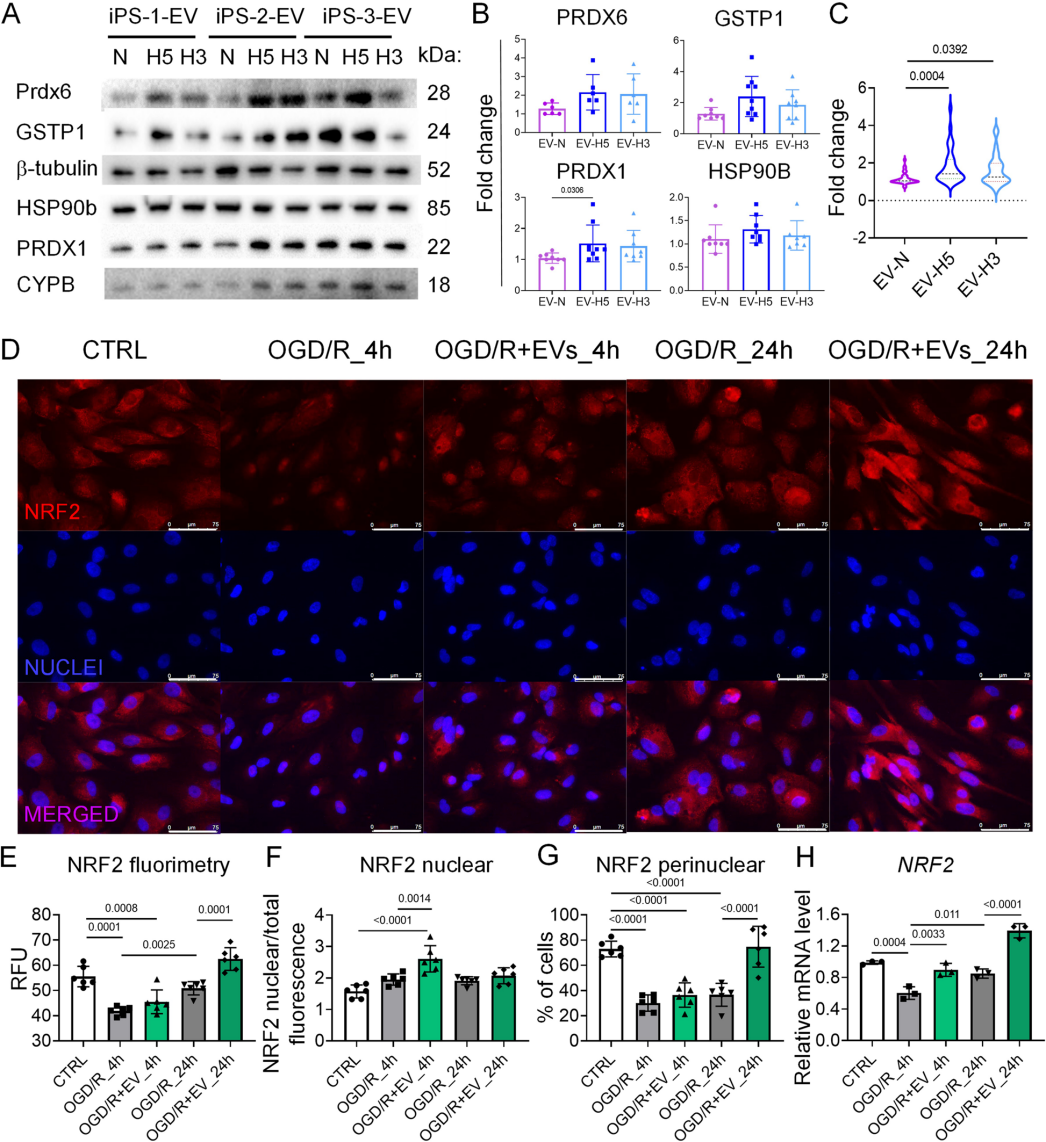
Analysis of protein levels associated with the NRF2-regulated pathway in hiPS-EVs and NRF2 transcription factor activity in human cardiomyocytes (CMs) subjected to oxygen-glucose deprivation followed by reoxygenation (OGD/R) and treated with hiPS-EVs derived from 5% oxygen (EV-H5, L-3). **A** Detection of selected antioxidant proteins in hiPS-EVs derived from three hiPSC lines cultured under different oxygen conditions (normoxia − 21% O_2_ - EV-N; hypoxia 5% O_2_ - EV-H5; hypoxia 3% O_2_ - EV-H3) by Western blot. Representative membranes are shown. **B** Densitometric measurement of antioxidant protein levels in hiPS-EVs, *n* = 6 (PRDX6) or *n* = 9 (GSTP1; PRDX1; HSP90B). **C** Cumulative analysis of the levels of proteins associated with the NRF2 signaling pathway in hiPS-EVs (PRDX6, GSTP1, PRDX1), *n* = 24. **D** Representative fluorescence images of NRF2 protein in hCMs after OGD/R and without or with EV-H5 treatment. Scale bar is 75 μm. Quantitative measurements of NRF2-related fluorimetry in cells (total fluorescence expressed in relative fluorescence units; RFU) (**E**), *n* = 6; or in nuclei (**F**), *n* = 6. **G **Percentage of cells expressing perinuclear localization of NRF2 protein, *n* = 6. **H **Analysis of *NRF2* transcript levels in CMs by real-time qPCR. *GAPDH* was used as an endogenous control, *n* = 3. All data are presented as mean ± SD. Statistical significance was tested by one-way ANOVA followed by Tukey’s post-hoc test (B: PRDX6; E-H), Kruskal-Wallis test followed by Dunn’s post-hoc test (B: GSTP1, PRDX1, HSP90B; C). Significant *p*-values (*p* < 0.05) are shown in each graph

We verified the presence of antioxidant proteins in hiPS-EVs identified in the proteomic analysis by Western blot (Fig. 7A, Additional File 12: Fig. S11). Densitometric analysis showed that all antioxidant proteins tested were upregulated under hypoxic conditions, with the highest abundance in EV-H5 (Fig. 7B, C). In particular, PRDX1 was significantly enriched in EV-H5 (relative protein level of 1.5 ± 0.6) compared to EV-N (protein level of 1 ± 0.2) (Fig. 7B). EV-H3 also contained an increased amount of PRDX6 protein (relative value of 1.4 ± 0.5), although the difference was not statistically significant. Overall, EVs derived from hypoxic conditions contained significantly higher levels of all antioxidant proteins tested from the NRF2 pathway (PRDX1, PRDX6 and GSTP1) compared to EV-N, in particular EV-H5 (Fig. 7C). To confirm the impact of EV-H5 on the NRF2 pathway in CMs, we performed additional studies based on the OGD/R assay of CM damage. Microscopic analysis of fluorescently labelled NRF2 protein showed an initial reduction in fluorescence signal in CMs subjected to OGD/R at 4 h post reoxygenation (41.9 ± 1.8 vs. 56.4 ± 3.8 RFU in control), which increased at a later time point (24 h post reoxygenation; reaching a value of 50.8 ± 2.9 RFU) (Fig. 7D, E). The signal was further enhanced by the addition of EV-H5 (45.5 ± 4.7 at 4 h post OGD/R and 62.5 ± 4.5 RFU at 24 h post OGD/R) (Fig. 7D, E). Knowing that NFR2 activity is mediated by its translocation to the nucleus, where it binds to antioxidant regulatory elements (ARE) and thereby regulates the expression of antioxidant proteins, we measured NRF2 fluorescence intensity in the nuclei of CMs. The ratio of nuclear to total fluorescence increased at 4 h post OGD/R compared to control (2 ± 0.1 vs. 1.5 ± 0.2) and remained unchanged at 24 h post OGD/R (2 ± 0.1) (Fig. 7F). The addition of therapeutic EV-H5 increased the nuclear localization of NRF2, particularly at 4 h after reoxygenation (the ratio of nuclear to total fluorescence was 2.4 ± 0.5), which then decreased slightly at 24 h (2.2 ± 0.1). Importantly, analysis of the fluorescence images also revealed an increased signal of NRF2 in the perinuclear region in CMs (Fig. 7G). The percentage of cells with perinuclear accumulation of NRF2 protein was highest in the control sample (73.4 ± 11.3%) and significantly decreased at 4 h after OGD/R (27.9 ± 9.3%). This value increased after treatment with EV-H5 (35.6 ± 12%), although not significantly. We observed a marked upregulation in the percentage of CMs with perinuclear localization of NRF2 at 24 h post OGD/R after the addition of EV-H5 (69.5 ± 20.5%, compared to 36.6 ± 13.7% for the sample without EVs) (Fig. 7G). The increase in NRF2 activity was further confirmed at the mRNA level. Transcription of the *NRF2* gene decreased after OGD/R insult (relative transcript level: 0.6 ± 0.1, compared to 1 ± 0.02 in the control), which was significantly increased after treatment with EV-H5 (0.9 ± 0.1) (Fig. 7H). A further increase in *NRF2* transcript level was observed 24 h after reoxygenation in the EV-H5 treated sample (relative mRNA value 1.4 ± 0.2) compared to untreated cells (0.85 ± 0.06).

**Fig. 8:**
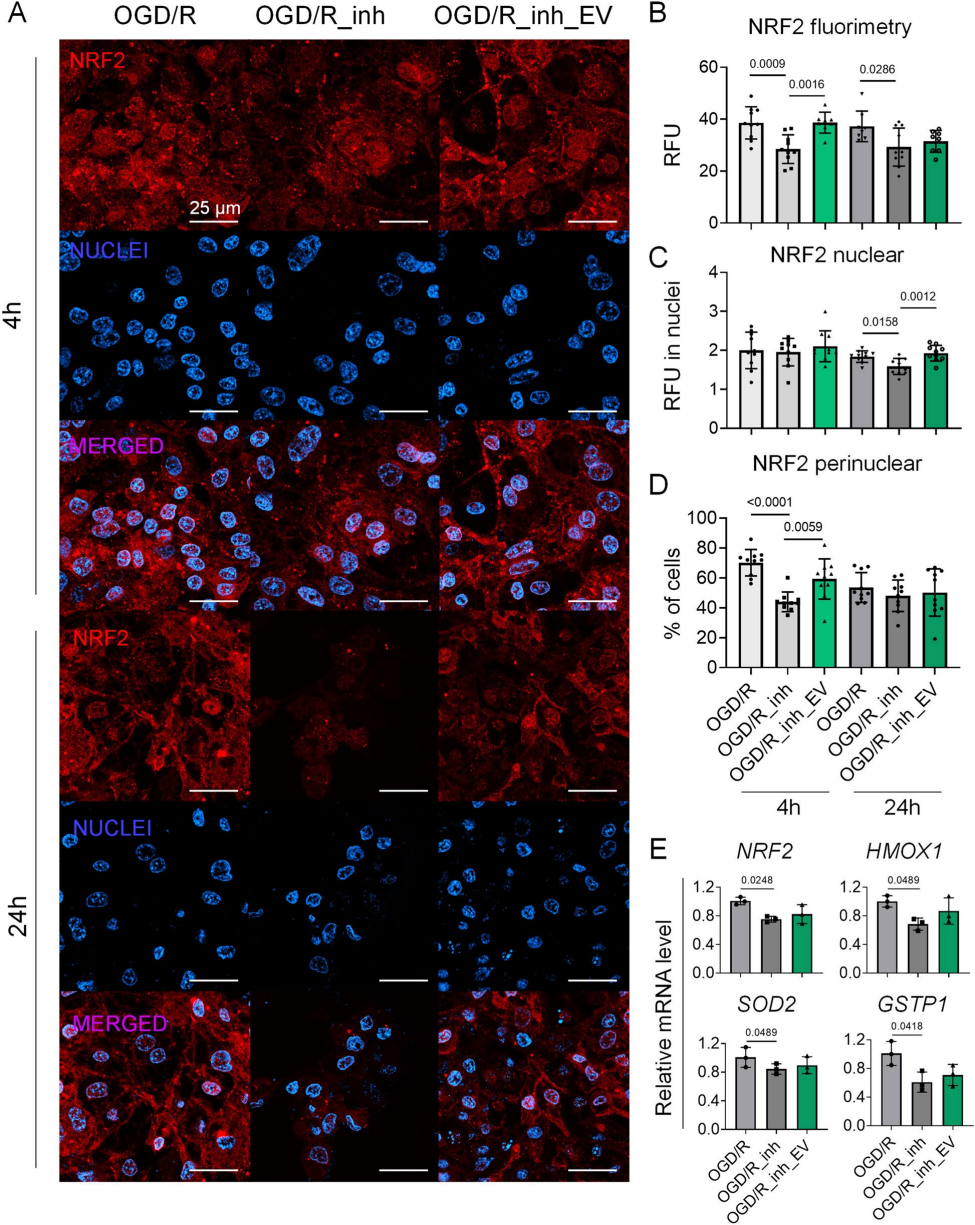
NRF2 inhibition in cardiomyocytes (CMs) subjected to oxygen-glucose deprivation followed by reoxygenation (OGD/R) and treated with hiPS-EVs derived from physiological hypoxia (5% O_2_; EV-H5, L-3). CMs were treated with NRF2 inhibitor ML385 (1 µM) for 4 and 24 h after reoxygenation, with or without addition of EVs. OGD/R-induced cells were used as controls. **A** Representative fluorescence images of NRF2 protein in hCMs after OGD/R, with or without ML385 or EV-H5 treatment. Scale bar is 25 μm. **B** Quantitative measurements of NRF2-related fluorimetry in cells (total fluorescence expressed in relative fluorescence units; RFU), *n* = 8–10; or in nuclei (**C**), *n* = 10. **D **Percentage of cells expressing perinuclear localization of NRF2 protein, *n* = 10. **E **Transcript levels of *NRF2* and NRF2-regulated genes (*HMOX1*, *SOD2*, *GSTP1*) in CMs by real-time qPCR, *n* = 3. All data are shown as mean ± SD. Statistical significance was tested by one-way ANOVA followed by Tukey’s post-hoc test. Significant *p*-values (*p* < 0.05) are shown in each graph

To gain a deeper insight into the EV-mediated induction of NRF2 activity in CMs, we used an NRF2 inhibitor ML385 in our OGD/R model. As the results show, the total level of NRF2 protein decreased in the presence of ML385 at both time points: 4 and 24 h after reoxygenation, compared to OGD/R-treated cells (28.5 ± 5.5 vs. 38.6 ± 6.2 RFU and 29.3 ± 7.3 vs. 37.2 ± 5.9 RFU, respectively) (Fig. 8A, B). The addition of EV-H5 partially rescued the cells 4 h after reoxygenation (38.7 ± 4 RFU). However, the level of NRF2 protein did not exceed the control level, which was even more pronounced 24 h after OGD/R treatment (31.5 ± 4.2 RFU). Similarly, nuclear localization of NRF2 decreased in the presence of ML385 24 h after reoxygenation (1.6 ± 0.2 RFU vs. 1.8 ± 0.15 RFU in control) and was restored after the treatment with EVs, but only to the control level (1.9 ± 0.2 RFU) (Fig. 8A, C). In contrast, the percentage of cells with perinuclear localization of NRF2 was significantly reduced in the presence of ML385 4 h after OGD/R (44 ± 6.6% vs. 70 ± 8.8% in control) and increased after the addition of EVs (59.2 ± 13.5%) (Fig. 8D). Importantly, the addition of ML385 reduced the transcript level of *NRF2* and its target genes (*HMOX1*, *SOD2* and *GSTP1*) and blunted the beneficial effect of EV-H5 measured 24 h after OGD/R (Fig. 8E).

## Discussion

The prevalence of CVDs continues to increase worldwide, in part due to the ageing and obesity of the human population. The postmitotic nature of the human heart, with its limited regenerative capacity, underscores the pressing need for innovative pro-regenerative strategies, presenting one of the greatest medical challenges of our time [1]. The permanent loss of CMs during ischemia/reperfusion necessitates the refinement of therapeutic targets to protect the heart from end-stage failure. Previous cell therapy approaches showed either a low or moderate level of cell engraftment and suggested that the transplanted cells’ paracrine effects are crucial mediators of the positive therapeutic outcomes [47]. Among the bioactive factors released by cells, EVs are increasingly being considered as next-generation therapeutics [48]. The role of EVs in tissue repair and regeneration is unparalleled. However, the direct mechanisms of EV-mediated activation of a specific cell type remain elusive. In particular, the question of which bioactive molecules encapsulated in EVs are primarily responsible for the observed functional effects has not been fully answered. Recent data suggest that protein cargo has the most determining role in EV-mediated function [49]. Therefore, in this study, we investigated the protein composition of hiPS-EVs obtained under different oxygen conditions (21, 5 and 3% O_2_) to reveal crucial molecules for cardio-protection.

Our mass spectrometry measurements revealed a large proportion of common proteins found in hiPS-EVs with a function related to metabolism of RNA and proteins (Additional File 4: Fig. S4). At the same time, EVs derived from specific oxygen conditions were enriched with proteins with distinct functions, which were identified by qualitative analysis. Similarly, differential protein expression analysis confirmed a significant effect of oxygen concentration in the cellular environment on the molecular composition of EVs. This finding aligns with other research, demonstrating that hypoxic preconditioning modulates EV cargo [20, 50–57]. We have shown that even relatively small difference in oxygen availability in the cell culture (3 vs. 5%) may induce vast changes in the EV molecular composition. Therefore, attention should be paid to careful designing of EV-tailored pro-regenerative treatment strategies.

To investigate the functional effects of hiPS-EVs derived from different oxygen concentrations, we used the well-established OGD/R model of CM injury. We demonstrated significantly reduced level of ROS, accompanied by enhanced transcription of antioxidant genes, particularly after EV-H5 (Fig. 3). To gain a deeper understanding of the molecular mechanisms underlying EV-mediated activity, we examined the state of the key signaling pathways that cross-react to salvage CMs upon injury. One such pathway, PI3K/Akt, plays a crucial role in protecting cells from death by synergistic potentiation of antioxidant, anti-inflammatory, and anti-apoptotic pathways [58]. A growing body of evidence demonstrates the protective effects of EV-driven activities of the PI3K/Akt signaling pathway against cardiac ischemia/reperfusion injury [13, 28, 51, 59]. Consistent with these observations, our findings indicate a pronounced Akt phosphorylation alongside a simultaneous reduction of its inhibitory counterpart PTEN, particularly 24 h post OGD/R insult (Fig. 4A, B). Exceptionally, EV-H5 exerted the most significant impact on CMs in comparison to EV-N, EV-H3 or EV-DF, confirming the beneficial influence of physiological hypoxia on the bioactive content of hiPS-EVs.

In order to maintain CMs homeostasis, crucial protein kinases work in conjunction with Akt to consolidate intracellular signaling cascades. Among these kinases, Erk1/2 is of primary importance [60]. In our study, both EV-N and EV-H5 substantially increased Erk1/2 phosphorylation, which contributed to overall CM survival (Fig. 4A, B). At 24 h post OGD/R, activation of Akt and Erk1/2 by hiPS-EVs counteracted the effects mediated by AMPK, as indicated by decreased phosphorylation of AMPK. Given that AMPK is a master energy sensor kinase that fulfils the metabolic demands of a cell during stress, its regulation in the ischemic heart is of great importance [61]. Studies have indicated that AMPK activation during ischemia leads to enhanced glucose utilization via translocation of vesicles containing glucose transporter type 4 (GLUT4) to the plasma membrane, thereby augmenting the glycolytic pathway and ATP production. Concomitantly, diminished levels of energy substrates initiate AMPK-mediated autophagy, which is vital for preserving energy homeostasis [61]. Notably, our differential protein expression analysis of hiPS-EVs revealed enhanced GLUT4 transfer to the cell surface by EV-H5 (Fig. 2), which corresponded to the initial upregulation of AMPK activity in CMs at 4 h post OGD/R (Additional file 2, Fig. 2). Enhanced AMPK signaling was observed in case of exosomes derived from MSCs, which alleviated myocardial ischemia/reperfusion injury [62]. However, elevated AMPK activity may only be clinically beneficial during ischemia or early reperfusion, as its overactivity may lead to excessive autophagy and loss of CMs [63]. Therefore, our OGD/R model has confirmed that the administration of hiPS-EVs led to a decrease in AMPK activity in CMs at 24 h post OGD/R (Fig. 4A, B), thereby preventing cell death.

Furthermore, EV-H5 significantly improved the functional properties of CMs. To demonstrate this, we used AFM-based spectroscopy and fluorescence microscopy, techniques whose application in cardiac research has not yet been fully recognized [64]. By measuring contractile parameters of CMs at the single cell level, we provide direct evidence of sustained contractility and calcium handling after EV-H5 treatment (Fig. 5).

In order to gain a deeper understanding of the molecular mechanisms behind the functional enhancement of CM activity induced by EV-H5, we focused on the most abundant proteins in EVs. Since direct transfer of EV cargo is one of several ways in which EVs induce changes in recipient cells, we hypothesized that only high-abundance molecules could surpass the threshold of molecular noise within a cell upon transfer, resulting in a significant change in cell fate. IBAQ and pathway enrichment analyses suggested NRF2-regulated signaling as a pathway differentially enriched in EV-H5 (Fig. 6). Importantly, the Western blot confirmed elevated levels of proteins, such as PRDX1, PRDX6 and GSTP1 in this pathway within EV-H5, as compared to EVs from other oxygen conditions (Fig. 7A-C). In contrast, the results of Gregorius J. et al. show a significant decrease in the PRDX6 protein in small EVs from MSC cultured under hypoxic conditions compared to those cultured under normoxic conditions [53]. However, the authors stimulated MSC with severe hypoxia at 1% O_2_. This confirms that EV-mediated biological effects are dependent on both the cell type and a specific level of oxygen. Fine-tuned regulation of oxygen availability in the cellular environment may shape the functional properties of the isolated EVs.

To dissect the results of the proteomic studies, we demonstrated enhanced NRF2 activity in CMs after EV-H5 treatment in the OGD/R model (Fig. 7D-H). Signaling from the NRF2 pathway has already been recognized as a critical step of the antioxidant defense mechanism in CMs against ischemia/reperfusion injury [25]. EVs can interfere with this process by either generating or scavenging ROS, thus potentiating or ameliorating oxidative damage [65]. A recent study showed that dysregulation of NRF2 signaling in infarcted hearts is caused by a set of specific miRNAs enriched in EVs released from injured cardiac tissue [66]. In contrast, enhanced antioxidant function and cardioprotection have been demonstrated for circulating EVs during exercise [67]. Improved reparative capacity through augmented NRF2 signaling has also been shown for MSC-derived EVs [27–30]. We have extended these data by demonstrating the antioxidant function of hiPS-EVs derived from physiological hypoxia. In this regard, we provide direct evidence for the involvement of hiPS-EVs in triggering the antioxidant response in injured CMs *via* the NRF2-regulated pathway. Importantly, we have shown that this signaling is abolished in CMs when co-incubated with the NRF2 inhibitor ML385 (Fig. 8). These data support the potential use of hypoxic hiPS-EVs for cardiac regeneration.

Although our study primarily focused on the protein cargo of hiPS-EVs, we cannot preclude the influence of other EV-enriched bioactive components that may play a role in cardioprotection, such as various RNA species and lipids. Notably, we and others have demonstrated that non-coding RNAs, including miRNAs, can affect the functionality of heart cells [13, 22]. Numerous miRNAs have been found to possess a pro-regenerative function and control pro-survival intracellular signaling pathways in heart tissue [68]. Our miRNA screening identified miR-302b-3p as the most abundant miRNA in hiPS-EVs with a strong anti-fibrotic function, which we confirmed in in vitro and in vivo studies [22]. Additionally, we recognized miR-19a-3p among the most abundant miRNAs in hiPS-EVs. Both of these miRNAs were shown to target PTEN [22, 69], which concomitantly increases Akt kinase activity and promotes cell survival. Importantly, the miR-302-367 cluster, which is associated with pluripotency, was found to be sufficient to induce CM proliferation and promote heart regeneration in mice [70]. These findings endorse the beneficial use of hiPS-EVs to convey miR-302 clusters and other bioactive molecules to recipient cells. It is thus more plausible that different entities encapsulated in EVs cooperatively impact intracellular pathways that regulate the function of target cells. Nevertheless, further study is needed to fully explore the therapeutic potential of EVs. This comprises evaluating the functionality of EVs obtained from diverse sources, defining their molecular cargo, and establishing reproducible protocols for isolation and clinical application in terms of optimal dosage and timing. This study makes a significant contribution to the rapidly developing field of EV-based innovative biomedicines by demonstrating that hiPSCs cultured under physiological hypoxia constitute a great source of therapeutic EVs.

## Conclusions

**Fig. 9:**
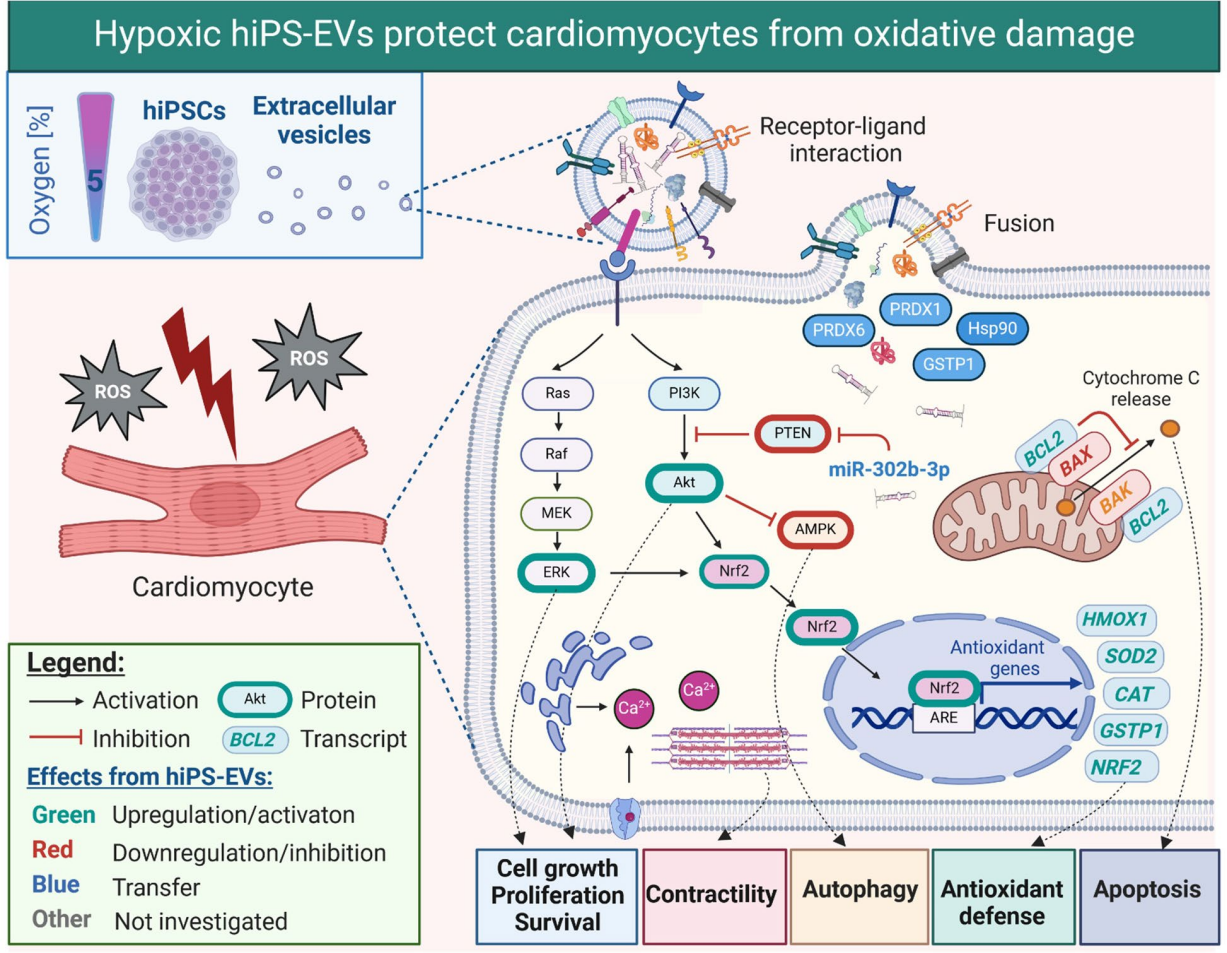
Summary of cytoprotective effects mediated by hiPS-EVs derived from physiological hypoxia (5% O_2_; EV-H5) on cardiomyocytes (CMs) subjected to oxygen-glucose deprivation followed by reoxygenation (OGD/R). EVs can affect CMs by interacting directly with cell surface receptors, thereby triggering intracellular signaling cascades, or by transferring their cargo upon fusion with the cell plasma membrane. Molecules abundant in EVs, including proteins of the NRF2-regulated antioxidant pathway (PRDX1, PRDX6, GSTP1) and miRNAs (such as miR-302b-3p), can enter CMs and play cytoprotective and regulatory roles. Intracellular molecular pathways regulated by EV-H5 include (1) cell growth, proliferation and survival; (2) contractility; (3) autophagy; (4) antioxidant defense; (5) apoptosis. The main effects observed at 24 h post OGD/R include: (1) significantly increased phosphorylation of Akt and Erk1/2 with concomitant downregulation of PTEN levels; (2) rescue of calcium flux and improved contractility; (3) downregulation of AMPK activity; (4) significantly increased transcription and translation of NRF2 with perinuclear accumulation of NRF2 protein; (5) significantly improved ratio of anti-apoptotic transcript *BCL2* to pro-apoptotic transcript *BAX* leading to increased cell survival

We have shown that hiPS-EVs obtained from various oxygen conditions, namely EV-N, EV-H5, and EV-H3, exhibit distinct proteomic signatures that influence their functional properties. All of them exerted beneficial effects on CMs, however, EV-H5 proved to be the most effective in triggering pro-survival pathways in CMs subjected to OGD/R. They outperformed hiPS-EVs from other oxygen conditions, as well as EVs derived from therapeutically inert cells - dermal fibroblasts (EV-DF). Not only did they efficiently support contractile activity and calcium signaling in damaged CMs, but they also contained the highest amount of antioxidant proteins, such as PRDX1, PRDX6, and GSTP1. We conclude that the cardioprotective effect of hiPS-EV, in particular EV-H5, is at least partly due to the synergistic activity of antioxidant proteins and miRNAs directly transferred from EVs to CMs, the enhancement of pro-survival kinases, including Akt and Erk1/2 and modulation of AMPK activity (Fig. 9). This induces a positive feedback loop leading to increased NRF2 expression and its nuclear activity, which helps to combat oxidative stress, rescues contractile function and protects CMs from apoptosis. Thus, the outcomes of this study hold promise for treating a range of pathological conditions with disturbed redox homeostasis, such as CVDs.

### Supplementary Information

Additional file 1: Figure S1. Western blot detection of hypoxia-inducible factors (HIFs) in three hiPSC lines (L1 – L3) used in this study. hiPSCs were cultured under different oxygen concentrations: ambient oxygen level (21% O_2_) – normoxia (N), or under reduced oxygen levels (hypoxia): at 5% O_2_ (H5) or 3% O_2_ (H3), for at least four passages. A. Images of Western blot membranes. B. Densitometric analysis of protein levels relative to control (*n*=3). C. Full size Western blot membranes shown in panel A. Data are presented as mean ± SD. Statistical significance was tested using ANOVA with the Tukey’s post-hoc test. Significant *p*-values (*p*<0.05) are shown in the graphs.

Additional file 2: Figure S2. Full size Western blot membranes of proteins typical of extracellular vesicles shown in main Figure 1C.

Additional file 3: Figure S3. Proteomic analysis of hiPSC-EVs. A. Number of proteins identified in each EV sample, based on at least two peptides. B. Venn diagrams showing common and distinct proteins in three EV samples from each group. Abbreviations: N – normoxia (21% O_2_); H5 – hypoxia at 5% O_2_; H3 – hypoxia at 3% O_2_.

Additional file 4: Figure S4. Pathways enrichment analysis of 1290 proteins identified as common in hiPS-EVs derived from different oxygen conditions: normoxia, hypoxia 5% O_2_ and hypoxia 3% O_2_ (detected in at least 6 out of 9 samples). The analysis was performed in the STRING web tool.

Additional file 5: Figure S5. Pathways enrichment analysis of distinct proteins identified in hiPS-EVs derived from different oxygen conditions: (A) normoxia (EV-N), (B) hypoxia 5% O_2_ (EV-H5) and (C) hypoxia 3% O_2_ (EV-H3), performed in the STRING web tool. The top 15 pathways with the most significant FDR value are shown for each EV type. The number of proteins included in the analysis is indicated in the subheading of each panel.

Additional file 6: Figure S6. Pathways analysis of differentially enriched proteins in EVs within the H3_N contrast using Ingenuity software. A graphical summary visualizing the predicted activation (orange) or inhibition (blue) is shown, along with the relationships between processes and proteins.

Additional file 7. Movie showing example of contracting cardiomyocytes derived from hiPS-L3 cell line used in this study.

Additional file 8: Figure S7. Gene expression analysis in hiPS-derived CMs (L3) by real-time qPCR. A. Comparison of transcript levels of genes characteristic for cardiomyocytes (*GATA4*, *TNNT2*), *n*=3 and (B) transcription factors regulating pluripotency (*OCT4* and *NANOG*), *n*=3. Statistical significance was tested using Student's T-test. Significant *p*-values (*p*<0.05) are shown in each graph.

Additional file 9: Figure S8. Effects of hiPS-EVs derived from three hiPSC lines cultured at different oxygen concentrations (21, 5 and 3% O_2_, designated EV-N, EV-H5 and EV-H3, respectively) and dermal fibroblast-derived EVs (EV-DF) on intracellular signaling pathways in cardiomyocytes (CMs). CMs were obtained by differentiation of hiPSCs-L3 and were subjected to oxygen-glucose deprivation followed by reoxygenation (OGD/R). Cells not treated with OGD/R were used as control (CTRL). The phosphorylation status and protein levels of selected proteins were analyzed 24 h post OGD/R. A. Western blot detection of activated kinases: Akt (Ser473), Erk1/2 (Thr202/Tyr204), AMPK (Thr172) and their total protein levels, as well as the total level of PTEN. GAPDH, and vinculin were used as controls. Representative membranes are shown. B. Densitometric analysis of protein levels detected by Western blot, relative to control, *n*=3-6. Statistical significance was tested using the Kruskal-Wallis test with Dunn's post-hoc test.

Additional file 10: Figure S9. Full size Western blot membranes of proteins shown in Figure S8.

Additional file 11: Figure S10. Full size Western blot membranes obtained in three independent experiments investigating the effect of hiPS-EVs derived from three hiPSC lines cultured under different oxygen concentrations (21, 5 and 3% O_2_) and dermal fibroblast-derived EVs on cardiomyocytes (CMs) in OGD/R model. Cells were analyzed 24 h after OGD/R insult. The following phosphorylation sites were detected: Akt (Ser473), Erk1/2 (Thr202/Tyr204), AMPK (Thr172). The membranes shown in the main figure (Fig. 4A) are indicated by blue rectangles with a dashed line.

Additional file 12: Figure S11. Full size Western blot membranes detecting selected antioxidant proteins in hiPS-EVs derived from three hiPSC lines cultured under different oxygen conditions (normoxia - 21% O_2_, hypoxia 5% O_2_ and 3% O_2_). The membranes shown in the main figure (Fig. 7A) are indicated by blue rectangles with a dashed line.

## Data Availability

The datasets used and/or analyzed during the current study are available from the corresponding author on reasonable request. Raw data from the proteomics studies are available at https://doi.org/10.57903/UJ/VZDNQW.

## Abbreviations

AFM: Atomic force microscopy
Akt: Protein kinase B
AMPK: Adenosine monophosphate-activated protein kinase
BSA: Bovine serum albumin
CAT: Catalase
CM: Cardiomyocyte
CPC: Cardiac progenitor cell
CVD: Cardiovascular disease
Erk1/2: Extracellular signal-regulated kinase 1/2
ESC: Embryonic stem cell
EV: Extracellular vesicle
EV-H3: Extracellular vesicles derived from hypoxic conditions at 3% O_2_
EV-H5: Extracellular vesicles derived from hypoxic conditions at 5% O_2_
EV-N: Extracellular vesicles derived from normoxic conditions at 21% O_2_
GST: Glutathione S-transferase
hiPS-EVs: human induced pluripotent stem cell-derived extracellular vesicles
HO-1: Heme oxygenase 1
HSP90B: Heat shock protein 90 alpha family class B member 1
iBAQ: Intensity-based absolute quantification
iPSC: induced pluripotent stem cell
LC-MS/MS: Liquid chromatography and tandem mass spectrometry
MSC: Mesenchymal stromal cell
NRF2: Nuclear factor (erythroid-derived 2)-like 2
OGD/R: Oxygen-glucose deprivation/reoxygenation
PRDX: Peroxiredoxin
PSC: Pluripotent stem cell
PTEN: Phosphatase and tensin homolog
qPCR: quantitative real-time polymerase chain reaction
ROS: Reactive oxygen species
SEC: Size exclusion chromatography
SOD2: Superoxide dismutase 2
UF: Ultrafiltration
