# Supplementary material for: Hypoxic extracellular vesicles from hiPSCs protect cardiomyocytes from oxidative damage by transferring antioxidant proteins and enhancing Akt/Erk/NRF2 signaling
Source: Cell Commun Signal. 2024 Jul 9;22:356. doi: 10.1186/s12964-024-01722-7 (PMC11232324; doi:10.1186/s12964-024-01722-7)
Supplement: Supplementary file 1 — Additional file 1: Figure S1. Western blot detection of hypoxia-inducible factors (HIFs) in three hiPSC lines (L1 – L3) used in this study. hiPSCs were cultured under different oxygen concentrations: ambient oxygen level (21% O2) – normoxia (N), or under reduced oxygen levels (hypoxia): at 5% O2 (H5) or 3% O2 (H3), for at least four passages. A. Images of Western blot membranes. B. Densitometric analysis of protein levels relative to control (n=3). C. Full size Western blot membranes shown in panel A. Data are presented as mean ± SD. Statistical significance was tested using ANOVA with the Tukey’s post-hoc test. Significant p-values (p<0.05) are shown in the graphs. [file 12964_2024_1722_MOESM1_ESM.pdf]

Additional File 1: Figure S1

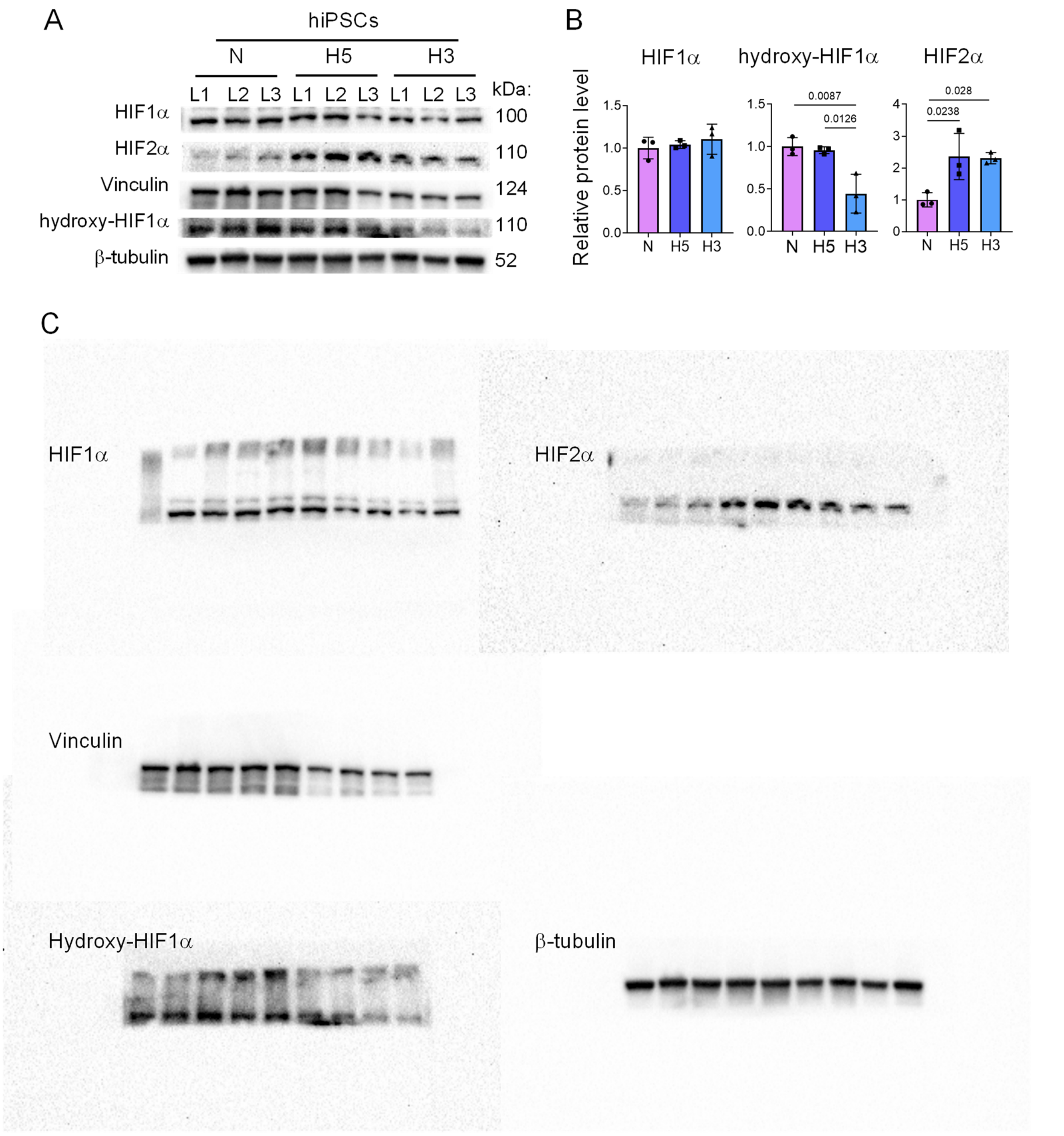

Figure S1. Western blot detection of hypoxia-inducible factors (HIFs) in three hiPSC lines (L1 – L3) used in this study. hiPSCs were cultured under different oxygen concentrations: ambient oxygen level (21% O_2) – normoxia (N), or under reduced oxygen levels (hypoxia): at 5% O_2 (H5) or 3% O_2 (H3), for at least four passages. **A.** Images of Western blot membranes. **B.** Densitometric analysis of protein levels relative to control (n=3). **C.** Full size Western blot membranes shown in panel A. Data are presented as mean ± SD. Statistical significance was tested using ANOVA with the Tukey's post-hoc test. Significant p-values ($p<0.05$) are shown in the graphs.
