# Supplementary material for: Hypoxic extracellular vesicles from hiPSCs protect cardiomyocytes from oxidative damage by transferring antioxidant proteins and enhancing Akt/Erk/NRF2 signaling
Source: Cell Commun Signal. 2024 Jul 9;22:356. doi: 10.1186/s12964-024-01722-7 (PMC11232324; doi:10.1186/s12964-024-01722-7)
Supplement: Supplementary file 2 — Additional file 2: Figure S2. Full size Western blot membranes of proteins typical of extracellular vesicles shown in main Figure 1C. [file 12964_2024_1722_MOESM2_ESM.pdf]

Additional File 2: Figure S2

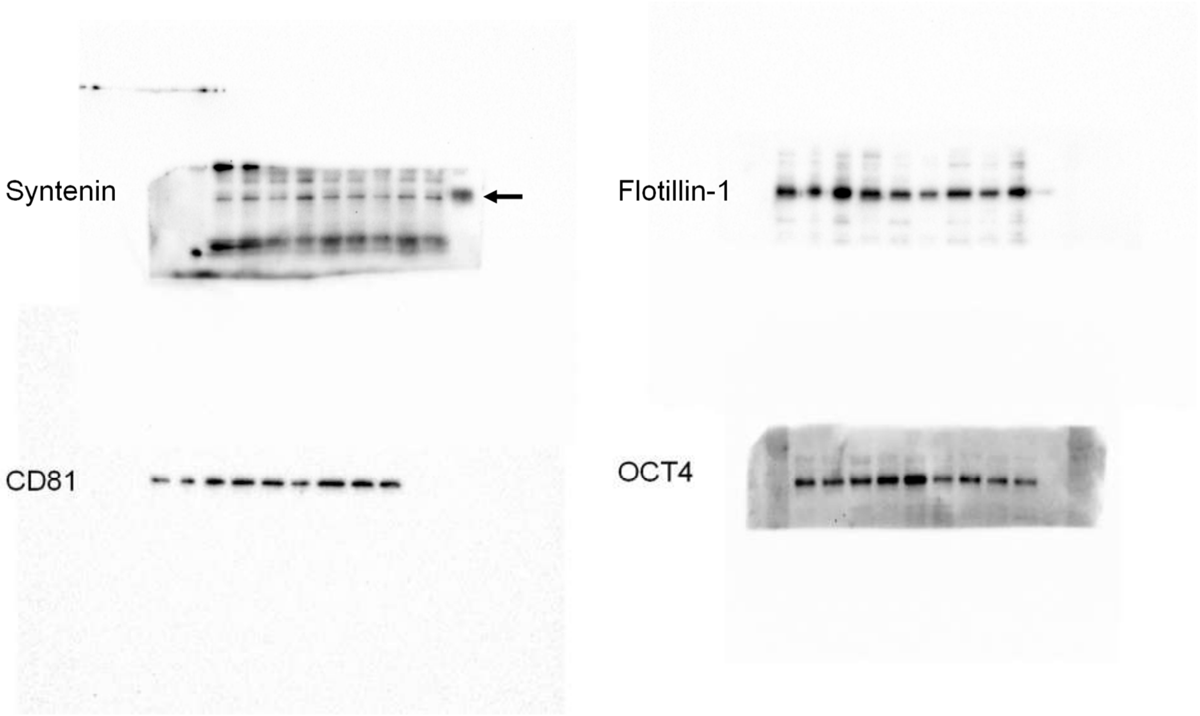

Figure S2. Full size Western blot membranes of proteins typical of extracellular vesicles shown in main Figure 1C.
