# Supplementary material for: Hypoxic extracellular vesicles from hiPSCs protect cardiomyocytes from oxidative damage by transferring antioxidant proteins and enhancing Akt/Erk/NRF2 signaling
Source: Cell Commun Signal. 2024 Jul 9;22:356. doi: 10.1186/s12964-024-01722-7 (PMC11232324; doi:10.1186/s12964-024-01722-7)
Supplement: Supplementary file 3 — Additional file 3: Figure S3. Proteomic analysis of hiPSC-EVs. A. Number of proteins identified in each EV sample, based on at least two peptides. B. Venn diagrams showing common and distinct proteins in three EV samples from each group. Abbreviations: N – normoxia (21% O2); H5 – hypoxia at 5% O2; H3 – hypoxia at 3% O2. [file 12964_2024_1722_MOESM3_ESM.pdf]

Additional File 3: Figure S3

A

Sample	#proteins	AVERAGE/SD	# non-redundant proteins in group
EVs_1_N	**2020**	**2081**	**2615**
EVs_2_N	**2165**	**75**	
EVs_3_N	**2057**		

Sample	#proteins	AVERAGE/SD	# non-redundant proteins in group
EVs_1_H5	**1904**	**1952**	**2408**
EVs_2_H5	**2069**	**102**	
EVs_3_H5	**1883**		

Sample	#proteins	AVERAGE/SD	# non-redundant proteins in group
EVs_1_H3	**1968**	**2042**	**2566**
EVs_2_H3	**2171**	**112**	
EVs_3_H3	**1986**		

B

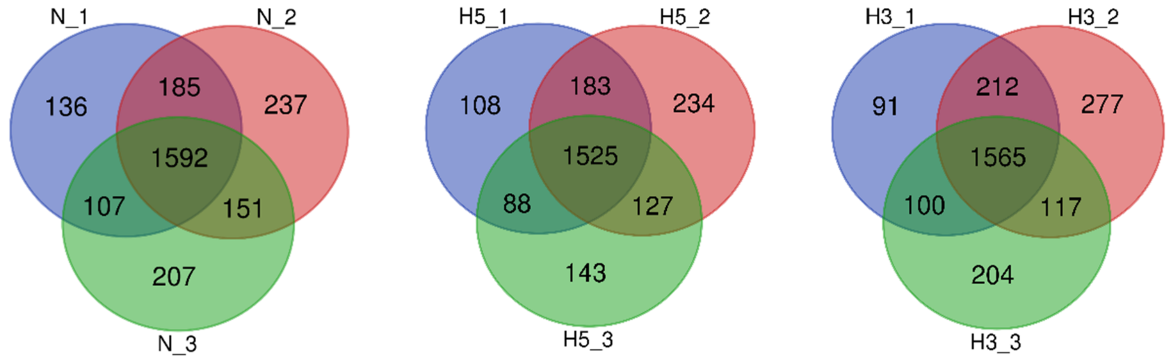

Figure S3. Proteomic analysis of hiPSC-EVs. **A.** Number of proteins identified in each EV sample, based on at least two peptides. **B.** Venn diagrams showing common and distinct proteins in three EV samples from each group. Abbreviations: N – normoxia (21% O_2); H5 – hypoxia at 5% O_2; H3 – hypoxia at 3% O_2.
