# Supplementary material for: Hypoxic extracellular vesicles from hiPSCs protect cardiomyocytes from oxidative damage by transferring antioxidant proteins and enhancing Akt/Erk/NRF2 signaling
Source: Cell Commun Signal. 2024 Jul 9;22:356. doi: 10.1186/s12964-024-01722-7 (PMC11232324; doi:10.1186/s12964-024-01722-7)
Supplement: Supplementary file 4 — Additional file 4: Figure S4. Pathways enrichment analysis of 1290 proteins identified as common in hiPS-EVs derived from different oxygen conditions: normoxia, hypoxia 5% O2 and hypoxia 3% O2 (detected in at least 6 out of 9 samples). The analysis was performed in the STRING web tool. [file 12964_2024_1722_MOESM4_ESM.pdf]

Additional File 4: Figure S4

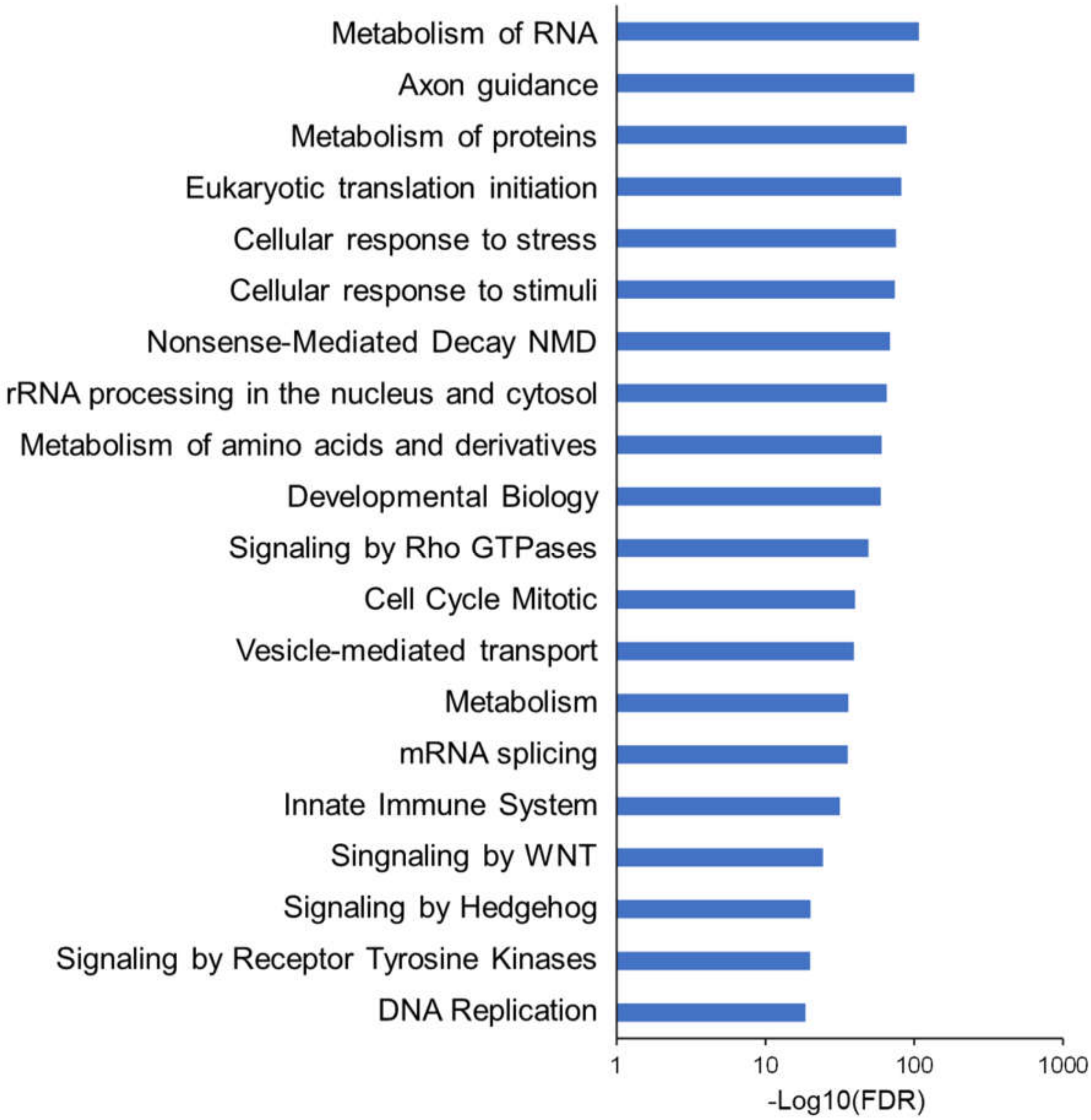

Figure S4. Pathways enrichment analysis of 1290 proteins identified as common in hiPS-EVs derived from different oxygen conditions: normoxia, hypoxia 5% O_2 and hypoxia 3% O_2 (detected in at least 6 out of 9 samples). The analysis was performed in the STRING web tool.
