# Supplementary material for: Hypoxic extracellular vesicles from hiPSCs protect cardiomyocytes from oxidative damage by transferring antioxidant proteins and enhancing Akt/Erk/NRF2 signaling
Source: Cell Commun Signal. 2024 Jul 9;22:356. doi: 10.1186/s12964-024-01722-7 (PMC11232324; doi:10.1186/s12964-024-01722-7)
Supplement: Supplementary file 5 — Additional file 5: Figure S5. Pathways enrichment analysis of distinct proteins identified in hiPS-EVs derived from different oxygen conditions: (A) normoxia (EV-N), (B) hypoxia 5% O2 (EV-H5) and (C) hypoxia 3% O2 (EV-H3), performed in the STRING web tool. The top 15 pathways with the most significant FDR value are shown for each EV type. The number of proteins included in the analysis is indicated in the subheading of each panel. [file 12964_2024_1722_MOESM5_ESM.pdf]

Additional File 5: Figure S5

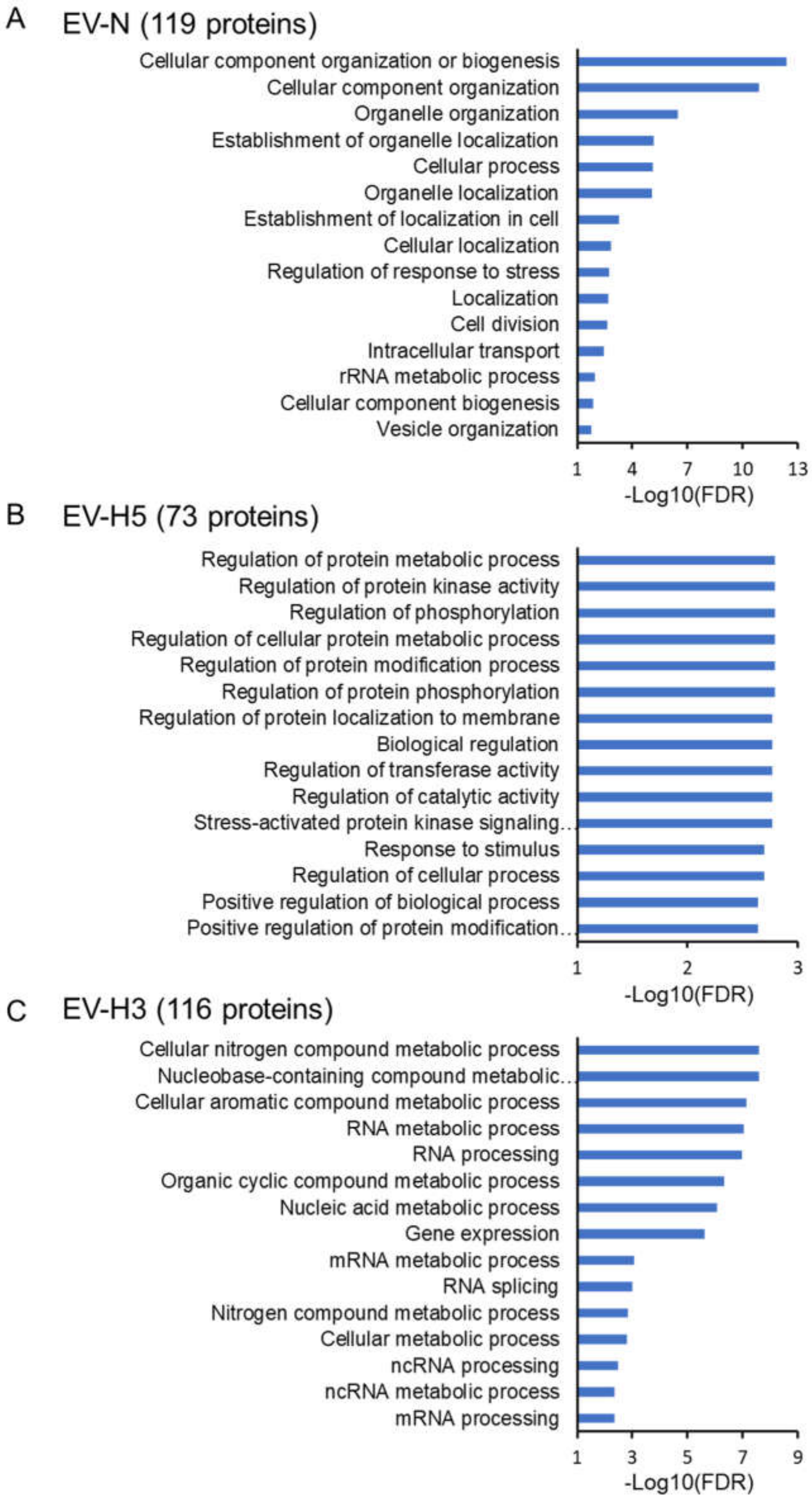

Figure S5. Pathways enrichment analysis of distinct proteins identified in hiPS-EVs derived from different oxygen conditions: (**A**) normoxia (EV-N), (**B**) hypoxia 5% O_2 (EV-H5) and (**C**) hypoxia 3% O_2 (EV-H3), performed in the STRING web tool. The top 15 pathways with the most significant FDR value are shown for each EV type. The number of proteins included in the analysis is indicated in the subheading of each panel.
