# Supplementary material for: Hypoxic extracellular vesicles from hiPSCs protect cardiomyocytes from oxidative damage by transferring antioxidant proteins and enhancing Akt/Erk/NRF2 signaling
Source: Cell Commun Signal. 2024 Jul 9;22:356. doi: 10.1186/s12964-024-01722-7 (PMC11232324; doi:10.1186/s12964-024-01722-7)
Supplement: Supplementary file 6 — Additional file 6: Figure S6. Pathways analysis of differentially enriched proteins in EVs within the H3_N contrast using Ingenuity software. A graphical summary visualizing the predicted activation (orange) or inhibition (blue) is shown, along with the relationships between processes and proteins. [file 12964_2024_1722_MOESM6_ESM.pdf]

Additional File 6: Figure S6

EV-H3_N – graphical abstract

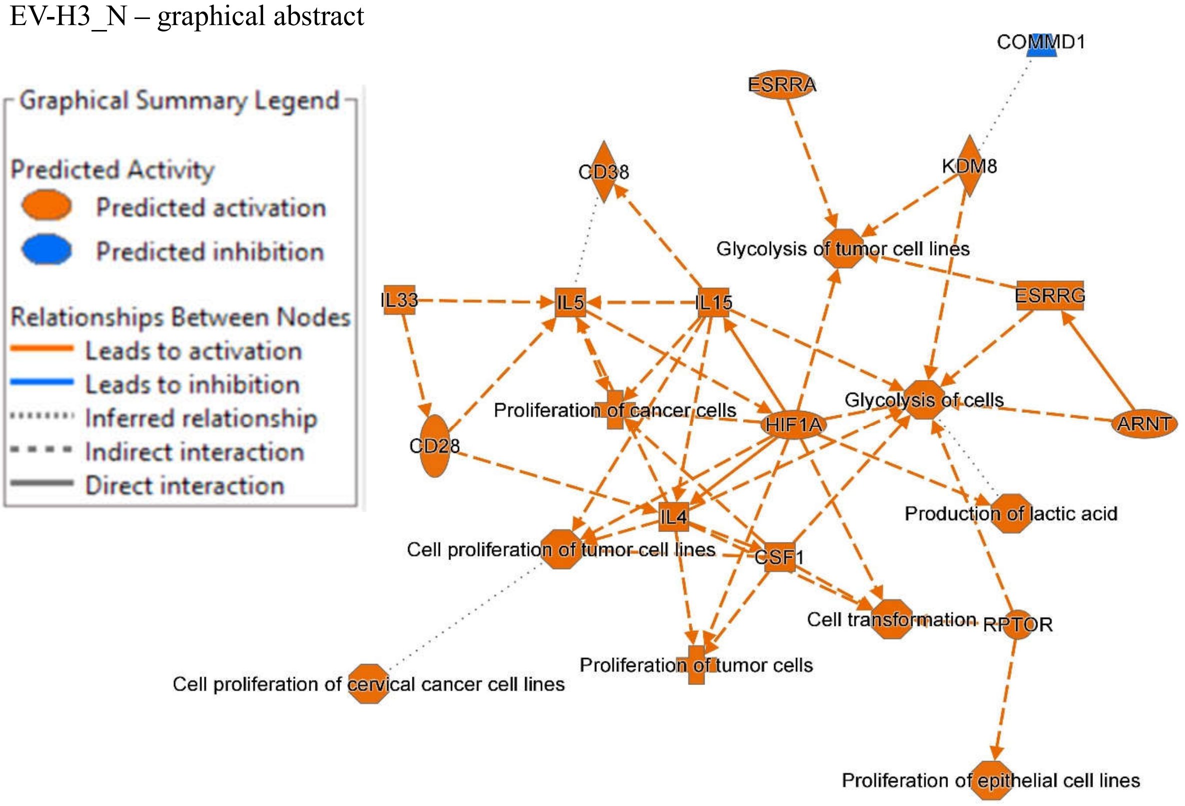

Figure S6. Pathways analysis of differentially enriched proteins in EVs within the H3_N contrast using Ingenuity software. A graphical summary visualizing the predicted activation (orange) or inhibition (blue) is shown, along with the relationships between processes and proteins.
