# Supplementary material for: Hypoxic extracellular vesicles from hiPSCs protect cardiomyocytes from oxidative damage by transferring antioxidant proteins and enhancing Akt/Erk/NRF2 signaling
Source: Cell Commun Signal. 2024 Jul 9;22:356. doi: 10.1186/s12964-024-01722-7 (PMC11232324; doi:10.1186/s12964-024-01722-7)
Supplement: Supplementary file 8 — Additional file 8: Figure S7. Gene expression analysis in hiPS-derived CMs (L3) by real-time qPCR. A. Comparison of transcript levels of genes characteristic for cardiomyocytes (GATA4, TNNT2), n=3 and (B) transcription factors regulating pluripotency (OCT4 and NANOG), n=3. Statistical significance was tested using Student's T-test. Significant p-values (p<0.05) are shown in each graph. [file 12964_2024_1722_MOESM8_ESM.pdf]

Additional File 8: Figure S7

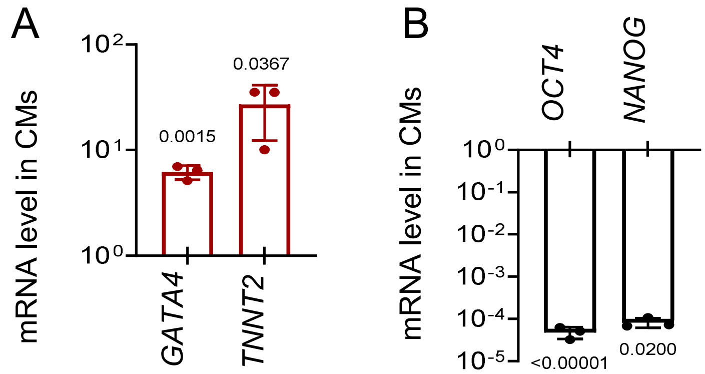

Figure S7. Gene expression analysis in hiPS-derived CMs (L3) by real-time qPCR. **A**. Comparison of transcript levels of genes characteristic for cardiomyocytes (*GATA4*, *TNNT2*), n=3 and (**B**) transcription factors regulating pluripotency (*OCT4* and *NANOG*), n=3. Statistical significance was tested using Student's T-test. Significant p-values ($p<0.05$) are shown in each graph.
