# Supplementary material for: Hypoxic extracellular vesicles from hiPSCs protect cardiomyocytes from oxidative damage by transferring antioxidant proteins and enhancing Akt/Erk/NRF2 signaling
Source: Cell Commun Signal. 2024 Jul 9;22:356. doi: 10.1186/s12964-024-01722-7 (PMC11232324; doi:10.1186/s12964-024-01722-7)
Supplement: Supplementary file 9 — Additional file 9: Figure S8. Effects of hiPS-EVs derived from three hiPSC lines cultured at different oxygen concentrations (21, 5 and 3% O2, designated EV-N, EV-H5 and EV-H3, respectively) and dermal fibroblast-derived EVs (EV-DF) on intracellular signaling pathways in cardiomyocytes (CMs). CMs were obtained by differentiation of hiPSCs-L3 and were subjected to oxygen-glucose deprivation followed by reoxygenation (OGD/R). Cells not treated with OGD/R were used as control (CTRL). The phosphorylation status and protein levels of selected proteins were analyzed 24 h post OGD/R. A. Western blot detection of activated kinases: Akt (Ser473), Erk1/2 (Thr202/Tyr204), AMPK (Thr172) and their total protein levels, as well as the total level of PTEN. GAPDH, and vinculin were used as controls. Representative membranes are shown. B. Densitometric analysis of protein levels detected by Western blot, relative to control, n=3-6. Statistical significance was tested using the Kruskal-Wallis test with Dunn's post-hoc test. [file 12964_2024_1722_MOESM9_ESM.pdf]

Additional File 9: Figure S8

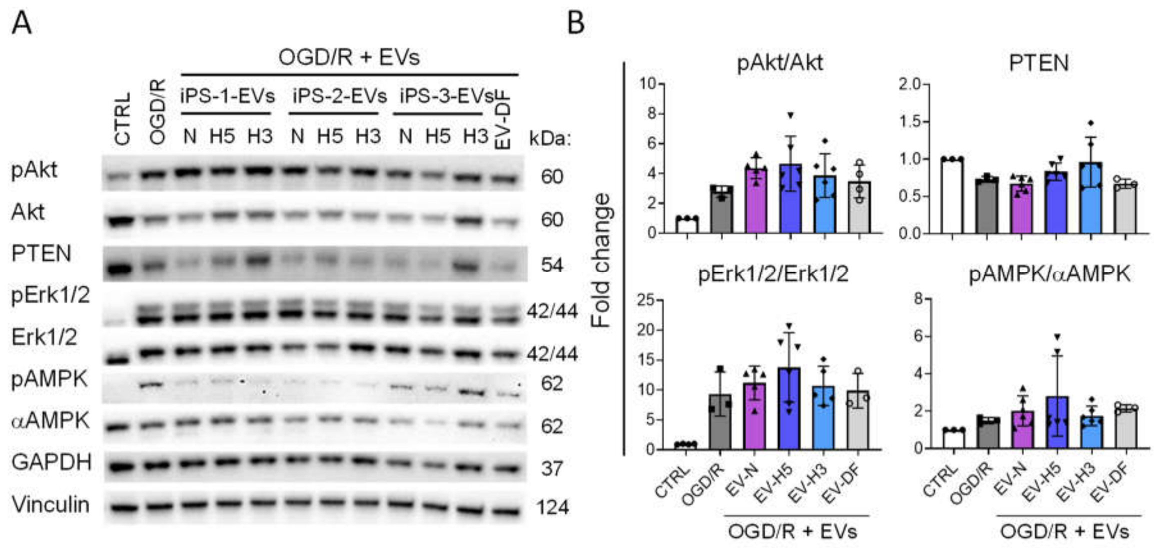

Figure S8. Effects of hiPS-EVs derived from three hiPSC lines cultured at different oxygen concentrations (21, 5 and 3% O_2, designated EV-N, EV-H5 and EV-H3, respectively) and dermal fibroblast-derived EVs (EV-DF) on intracellular signaling pathways in cardiomyocytes (CMs). CMs were obtained by differentiation of hiPSCs-L3 and were subjected to oxygen-glucose deprivation followed by reoxygenation (OGD/R). Cells not treated with OGD/R were used as control (CTRL). The phosphorylation status and protein levels of selected proteins were analyzed 24 h post OGD/R. **A.** Western blot detection of activated kinases: Akt (Ser473), Erk1/2 (Thr202/Tyr204), AMPK (Thr172) and their total protein levels, as well as the total level of PTEN. GAPDH, and vinculin were used as controls. Representative membranes are shown. **B.** Densitometric analysis of protein levels detected by Western blot, relative to control, n=3-6. Statistical significance was tested using the Kruskal-Wallis test with Dunn's post-hoc test.
