# Supplementary material for: Hypoxic extracellular vesicles from hiPSCs protect cardiomyocytes from oxidative damage by transferring antioxidant proteins and enhancing Akt/Erk/NRF2 signaling
Source: Cell Commun Signal. 2024 Jul 9;22:356. doi: 10.1186/s12964-024-01722-7 (PMC11232324; doi:10.1186/s12964-024-01722-7)
Supplement: Supplementary file 10 — Additional file 10: Figure S9. Full size Western blot membranes of proteins shown in Figure S8. [file 12964_2024_1722_MOESM10_ESM.pdf]

**Additional File 10: Figure S9**

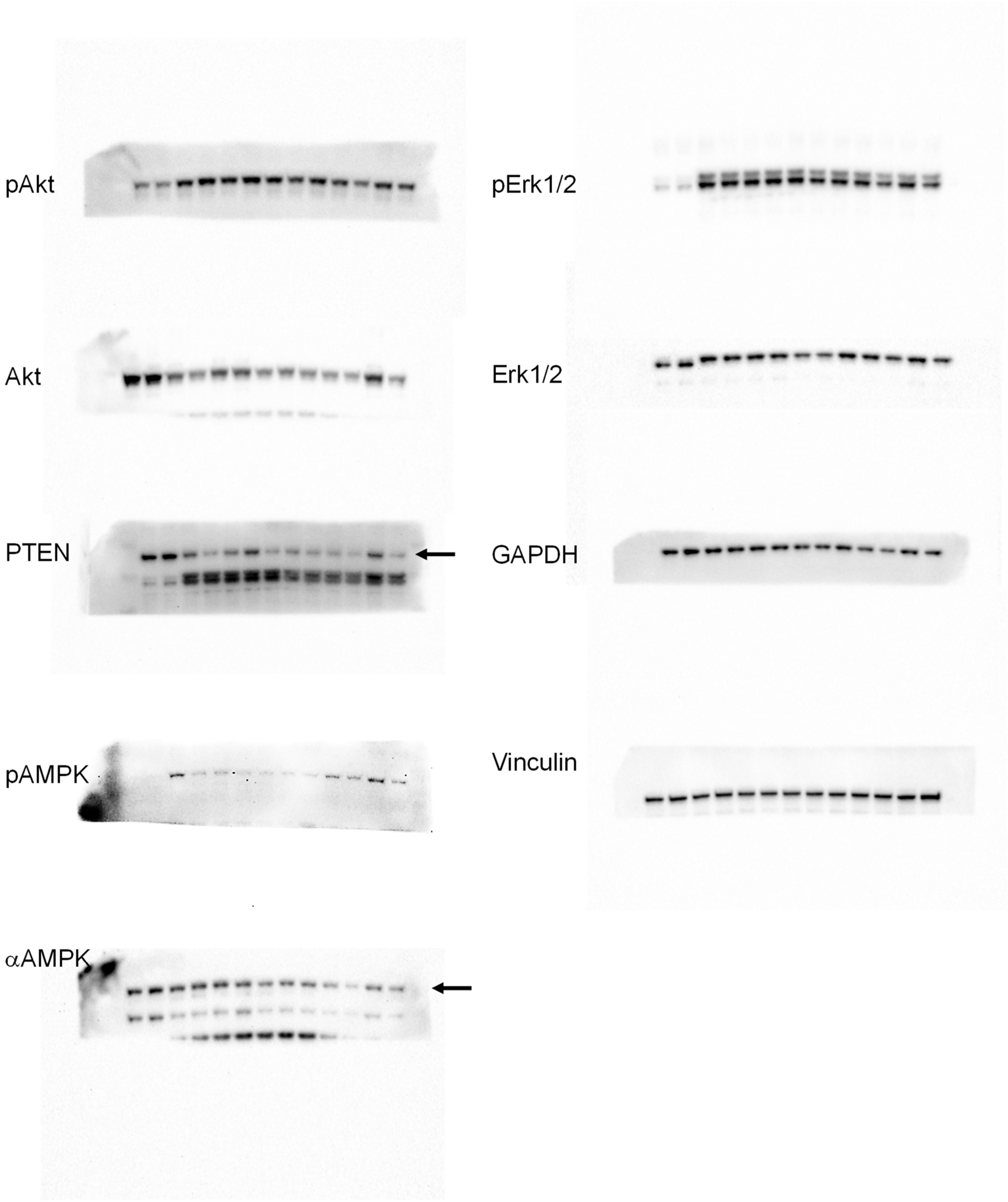


**Figure S9**. Full size Western blot membranes of proteins shown in Figure S8.
