# Supplementary material for: Hypoxic extracellular vesicles from hiPSCs protect cardiomyocytes from oxidative damage by transferring antioxidant proteins and enhancing Akt/Erk/NRF2 signaling
Source: Cell Commun Signal. 2024 Jul 9;22:356. doi: 10.1186/s12964-024-01722-7 (PMC11232324; doi:10.1186/s12964-024-01722-7)
Supplement: Supplementary file 11 — Additional file 11: Figure S10. Full size Western blot membranes obtained in three independent experiments investigating the effect of hiPS-EVs derived from three hiPSC lines cultured under different oxygen concentrations (21, 5 and 3% O2) and dermal fibroblast-derived EVs on cardiomyocytes (CMs) in OGD/R model. Cells were analyzed 24 h after OGD/R insult. The following phosphorylation sites were detected: Akt (Ser473), Erk1/2 (Thr202/Tyr204), AMPK (Thr172). The membranes shown in the main figure (Fig. 4A) are indicated by blue rectangles with a dashed line. [file 12964_2024_1722_MOESM11_ESM.pdf]

Additional File 11: Figure S10

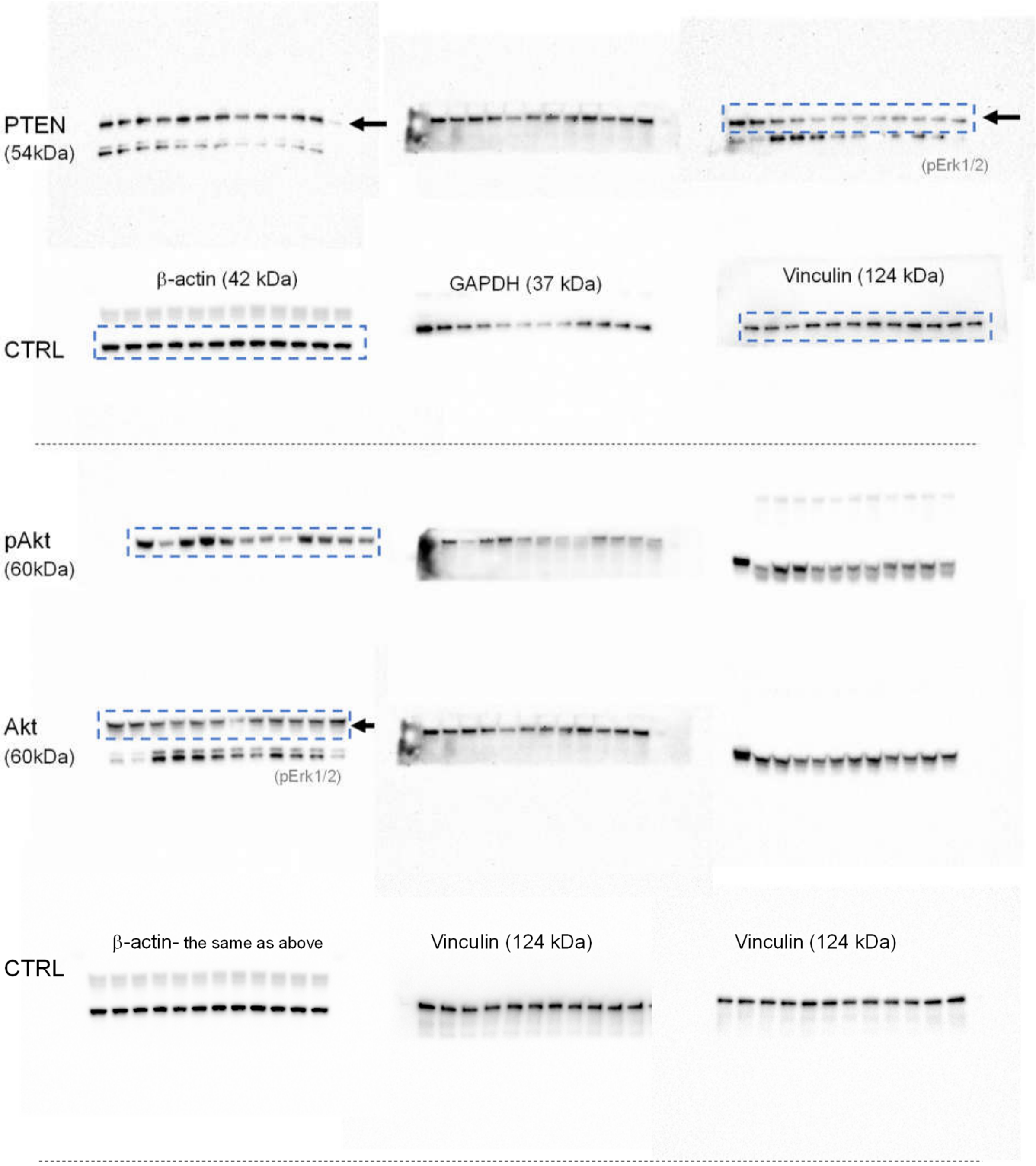

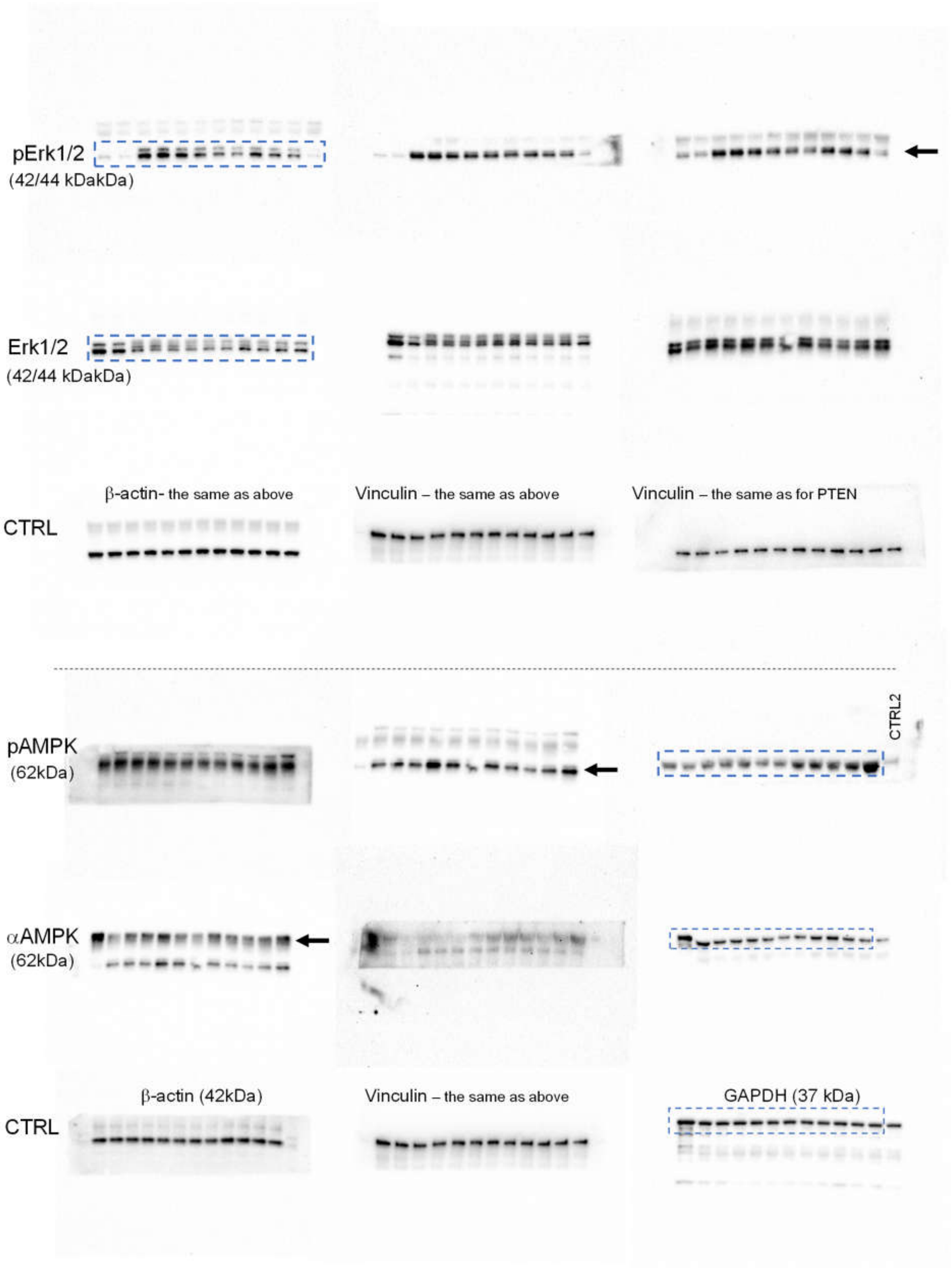

Figure S10. Full size Western blot membranes obtained in three independent experiments investigating the effect of hiPS-EVs derived from three hiPSC lines cultured under different oxygen concentrations (21, 5 and 3% O2) and dermal fibroblast-derived EVs on cardiomyocytes (CMs) in OGD/R model. Cells were analyzed 24 h after OGD/R insult. The following phosphorylation sites were detected: Akt (Ser473), Erk1/2 (Thr202/Tyr204), AMPK (Thr172). The membranes shown in the main figure (Fig. 4A) are indicated by blue rectangles with a dashed line.
