# Supplementary material for: Hypoxic extracellular vesicles from hiPSCs protect cardiomyocytes from oxidative damage by transferring antioxidant proteins and enhancing Akt/Erk/NRF2 signaling
Source: Cell Commun Signal. 2024 Jul 9;22:356. doi: 10.1186/s12964-024-01722-7 (PMC11232324; doi:10.1186/s12964-024-01722-7)
Supplement: Supplementary file 12 — Additional file 12: Figure S11. Full size Western blot membranes detecting selected antioxidant proteins in hiPS-EVs derived from three hiPSC lines cultured under different oxygen conditions (normoxia - 21% O2, hypoxia 5% O2 and 3% O2). The membranes shown in the main figure (Fig. 7A) are indicated by blue rectangles with a dashed line. [file 12964_2024_1722_MOESM12_ESM.pdf]

**Additional File 12: Figure S11**

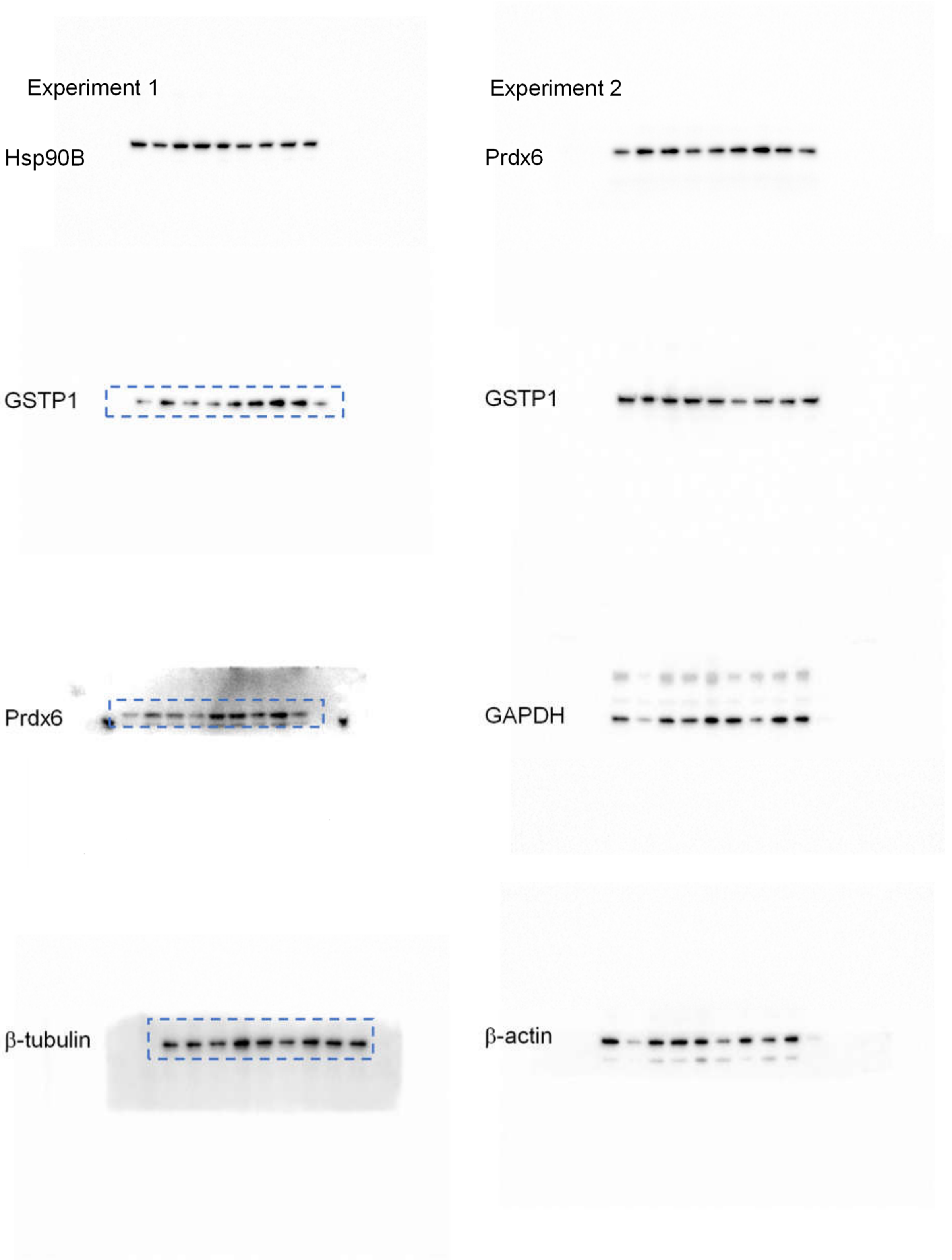

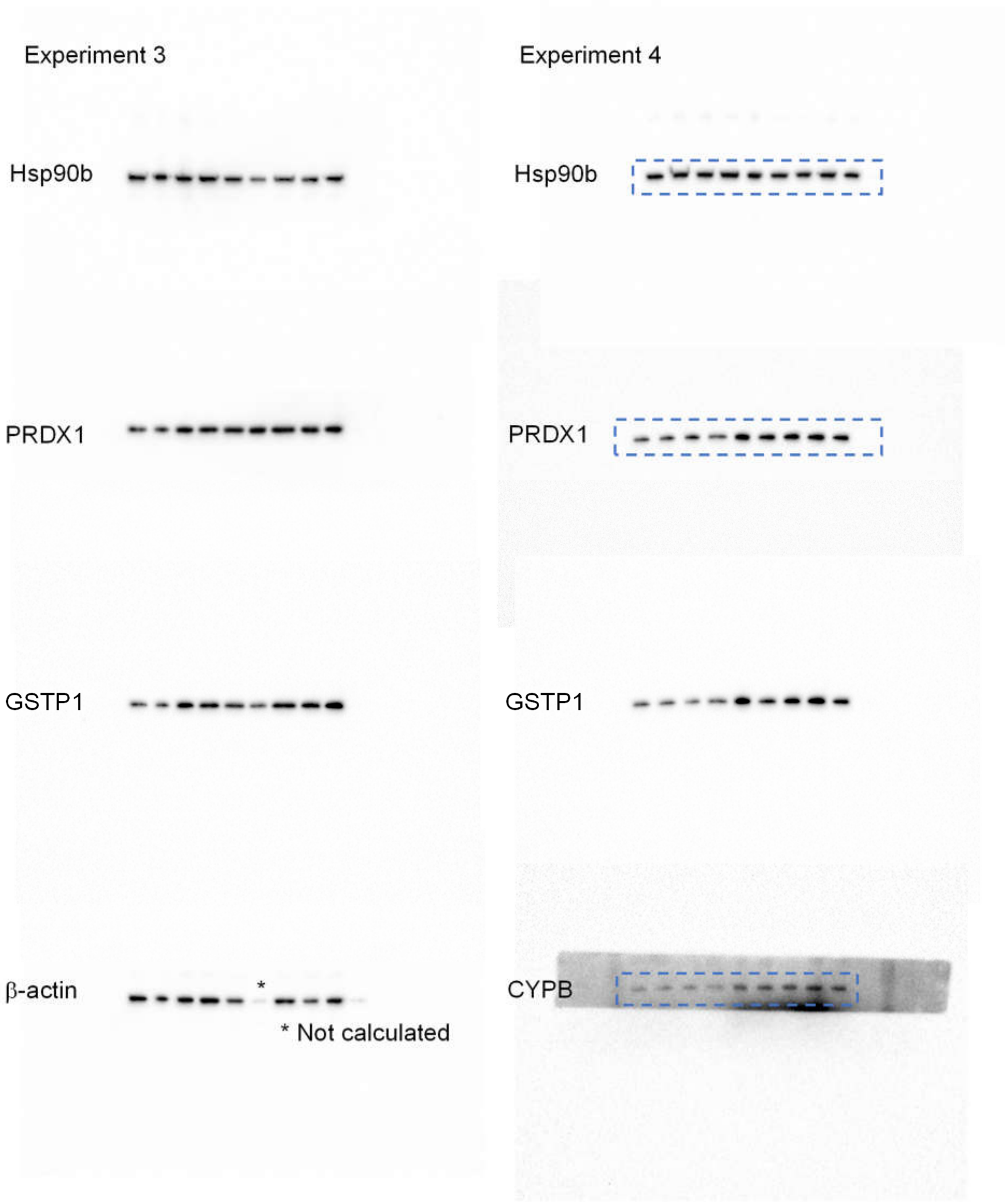


**Figure S11.** Full size Western blot membranes detecting selected antioxidant proteins in hiPS-EVs derived from three hiPSC lines cultured under different oxygen conditions (normoxia - 21% $O_2$, hypoxia 5% $O_2$ and 3% $O_2$). The membranes shown in the main figure (Fig. 7A) are indicated by blue rectangles with a dashed line.
